# Redirecting Cholesterol Metabolism During *Ex Vivo* Biomanufacturing Enhances the Metabolic Fitness and Antitumor Efficacy of CAR-T Cells

**DOI:** 10.7150/ijbs.132207

**Published:** 2026-05-11

**Authors:** Yuge Zhu, Jiaxin Tu, Shuang Li, Shance Li, Bufan Xiao, Xuantong Zhou, Guanyu Zhang, Xinyu Li, You He, Nan Wu, Zheming Lu, Chaoting Zhang

**Affiliations:** 1 Key Laboratory of Carcinogenesis and Translational Research (Ministry of Education/Beijing), Department of Thoracic Surgery II, Peking University Cancer Hospital & Institute, Beijing 100142, China.; 2 Key Laboratory of Carcinogenesis and Translational Research (Ministry of Education/Beijing), Laboratory of Biochemistry and Molecular Biology, Peking University Cancer Hospital & Institute, Beijing, 100142, China.; 3 The school of artificial intelligence, Beihang University, Beijing, China.; 4 State Key Laboratory of Molecular Oncology, Frontiers Science Center for Cancer Integrative Omics, Department of Thoracic Surgery II, Beijing Key Laboratory of Carcinogenesis and Translational Research, Peking University Cancer Hospital & Institute, Beijing, 100142, China.; 5 Yunnan Cancer Hospital, The Third Affiliated Hospital of Kunming Medical University, Peking University Cancer Hospital Yunnan, Yunnan, China.

**Keywords:** CAR-T cells, cholesterol, low-dose FL, metabolic remodeling

## Abstract

Chimeric antigen receptor T (CAR-T) cell therapy has revolutionized the treatment of hematologic malignancies; however, its efficacy in solid tumors remains limited, partly due to T cell exhaustion during *ex vivo* manufacturing. Emerging evidence suggests that cholesterol metabolism plays a critical role in T cell differentiation and function, yet its impact during CAR-T cell production is poorly understood. We investigated the effects of cholesterol modulation during* ex vivo* CAR-T cell expansion by using low-dose fluvastatin (FL), a clinically approved HMG-CoA reductase inhibitor. We found that cholesterol accumulation during *ex vivo* expansion promotes CAR-T cell exhaustion. Low-dose FL reduces cholesterol to physiological levels, preserving a less-differentiated, memory-enriched phenotype and attenuating exhaustion, thereby enhancing CAR-T cell cytotoxicity and persistence without affecting viability. In multiple xenograft models, FL-primed CAR-T cells demonstrate superior *in vivo* expansion, persistence, and tumor control. Mechanistically, FL enhances ERK_1/2_ phosphorylation to remodel CAR-T cell metabolism from glycolysis to oxidative phosphorylation. Inhibiting ERK_1/2_ or ATP synthesis abrogates these benefits, indicating that ERK_1/2_-dependent mitochondrial metabolism is required for CAR-T cell functional improvements conferred by FL. These findings establish cholesterol metabolism as a tunable axis during CAR-T cell manufacturing and propose a clinically feasible, GMP-compatible strategy to enhance CAR-T cell fitness and therapeutic efficacy.

## Introduction

Chimeric antigen receptor T (CAR-T) cell therapy has revolutionized the treatment landscape of hematologic malignancies and holds promise for solid tumors 1-3. However, challenges such as limited *in vivo* persistence, functional exhaustion, and terminal differentiation continue to hinder broader clinical success 4, 5. Substantial evidence suggests that CAR-T cells enriched in naïve and central memory subsets, with low exhaustion levels, exhibit superior expansion, persistence, and antitumor activity in patients 6, 7. Accordingly, strategies to preserve or enhance T cell functionality during the *ex vivo* manufacturing process are of critical importance 8-11.

Metabolic reprogramming has emerged as a key determinant of T cell fate and function 12, 13. Cholesterol metabolism, in particular, has been implicated in T cell activation, differentiation, and immune responses 14, 15. During *ex vivo* expansion, T cells undergo metabolic shifts, including enhanced cholesterol biosynthesis, to support rapid proliferation and membrane synthesis demands 16, 17. While elevated cholesterol in T cells or the TME, has been reported to enhance TCR signaling and promote effector responses in acute settings, excessive cholesterol accumulation can trigger ER stress and promote terminal exhaustion—especially under chronic stimulation or prolonged expansion 17-19. On the other hand, cholesterol depletion impairs T cell proliferation and membrane biosynthesis 15. This dichotomy underscores the importance of maintaining cholesterol homeostasis within an optimal range during CAR-T cell manufacturing, which is likely critical for ensuring final product quality and therapeutic efficacy.

In this study, we investigated whether modulating cholesterol metabolism through fluvastatin (FL), a competitive HMG-CoA reductase inhibitor, could improve CAR-T cell phenotype and function. Through dose titration, we identified that low-dose FL was sufficient to enhance CAR-T cell phenotype and function without compromising viability or expansion. In contrast, higher doses led to reduced proliferation and survival, likely due to excessive inhibition of cholesterol biosynthesis. These results underscore the importance of maintaining cholesterol homeostasis and support finely tuned metabolic modulation to optimize CAR-T cell manufacturing. Mechanistically, low-dose FL reprogrammed CAR-T cell metabolism from glycolysis toward mitochondrial oxidative phosphorylation (OXPHOS) via ERK_1/2_ activation—a shift essential for preserving memory-like characteristics and functional potential. Together, our findings suggest that moderate modulation of cholesterol metabolism during *ex vivo* manufacturing—specifically through low-dose FL—can improve CAR-T cell phenotype and function, offering a promising strategy to enhance the therapeutic potential of CAR-T cells.

## Methods

### Cell Lines and Culture Conditions

The Burkitt lymphoma cell line Raji, erythroleukemia cell line K562, and non-small cell lung cancer (NSCLC) cell lines A549 and H1299 were obtained from the American Type Culture Collection (ATCC). All cell lines were cultured in RPMI-1640 medium (Gibco) supplemented with 10% fetal bovine serum (FBS; Sigma-Aldrich) in a humidified incubator at 37°C with 5% CO₂. K562 and H1299 cells were stably transduced with lentiviral vectors encoding human CD19 and mesothelin (MSLN), respectively. Raji and A549 cells were transduced with lentiviral vectors co-expressing green fluorescent protein (GFP) and firefly luciferase for *in vivo* imaging. Primary human T cells were isolated from peripheral blood of healthy donors with informed consent. T cells were activated for 3 days in X-VIVO15 medium (Lonza) supplemented with 50 ng/mL anti-CD3 (ACRO Biosystems), 1 μg/mL anti-CD28 (TLBiotechnology), and 5 ng/mL IL-2 (PeproTech).

### Lentiviral Packaging and CAR-T Cell Generation

Lentiviral vectors encoding CD19 or MSLN CAR constructs were co-transfected into 293FT cells with packaging and envelope plasmids using polyethylenimine (PEI; Polysciences), as previously described 20. Supernatants were harvested at 48 and 72 hours post-transfection, filtered (0.45 μm, Millipore), and concentrated via ultracentrifugation. Activated T cells were transduced with the concentrated lentivirus in the presence of 8 μg/mL polybrene (Sigma-Aldrich). Transduction efficiency was assessed 72 hours later by flow cytometry.

### Flow Cytometry

T cells were washed with cold 1×DPBS containing 1% BSA (Sigma-Aldrich). T cells were first stained with FVS 780 dye at room temperature in the dark for 15 minutes before analysis. Surface staining was performed at 4°C for 30 minutes in the dark using the following antibodies: BUV395-anti-CD3 and FITC-anti-FLAG (transduction), BUV737-anti-PD-1, APC-anti-LAG-3, PE-anti-TIM-3 (exhaustion), PE-anti-CCR7, and APC-anti-CD45RA (memory phenotype; BD Biosciences). Intracellular staining of Ki67 (AF647; BD Biosciences) was performed after fixation and permeabilization using BD Cytofix/Cytoperm buffer. For cholesterol content analysis, cells were fixed with 4% paraformaldehyde (PFA; Solarbio) and stained with 50 μg/mL Filipin III (GLPBIO) at room temperature for 1 hour in the dark. For analysis of mitochondrial mass and membrane potential, cells were washed and incubated in X-VIVO 15 medium containing either MitoTracker Green FM (for mitochondrial mass; Thermo Fisher Scientific) or MitoProbe™ TMRM (for membrane potential; Thermo Fisher Scientific) at 37 °C for 15 minutes in the dark. Samples were analyzed on a BD FACS Aria II cytometer and processed using FlowJo v10 software.

### Cytokine Quantification

CAR-T cells (1×10⁵) were co-cultured with tumor cells (5×10⁴) for 24 hours. Supernatants were collected and assayed for IFN-γ, IL-2, and TNF-α using ELISA kits (R&D Systems) per the manufacturer's instructions.

### *In Vitro* Cytotoxicity Assay

Tumor cells (5×10⁴) were labeled with 5 μM CFSE (BD Biosciences) and co-cultured with effector CAR-T cells at various E:T ratios (1:2 to 4:1 for CD19 CAR-T cells; 1:1 to 8:1 for MSLN CAR-T cells) for 6-8 hours. Cells were stained with propidium iodide (PI; BD Biosciences) and analyzed on an Accuri C6 flow cytometer.

### Chronic antigen exposure assay

A549 cells were seeded in 24-well plates at 3×10^5^/well and pretreated with 20 μg/mL mitomycin C for 2 h to prevent proliferation while preserving their antigen expression and short-term stimulatory capacity. Subsequently, MSLN or FL-primed MSLN CAR-T cells were added at an E:T ratio of 1:2 and co-cultured with A549 for 3 days. Similarly, Raji cells were routinely seeded in poly-L-lysine-coated 24-well plates at 8×10^5^/well and pretreated with 10 μg/mL mitomycin C for 2 h, followed by the addition of CD19 CAR-T cells or FL-primed CD19 CAR-T cells. After each round of tumor cell rechallenge, the CAR-T cells were harvested and used for cytokine and cytotoxicity analyses and then re-exposed to fresh tumor cells.

### *In Vivo* Xenograft Models

All animal experiments were approved by the Institutional Animal Care and Use Committee (IACUC) of Beijing Cancer Hospital. Female NCG mice (6-8 weeks old; JC Bioscience) were maintained under specific pathogen-free conditions. For the hematologic model, mice were intravenously injected with Raji-luc cells (1×10⁶). Four days later, mice were randomized by tumor burden (bioluminescence, BLI) and treated with MOCK, control, or FL-primed CD19 CAR-T cells (1×10⁶). Tumor progression was monitored using bioluminescence imaging (BLI; PerkinElmer). Peripheral blood was collected under inhalation anesthesia on day 6 and 14 post-treatment for T cell exhaustion and memory phenotype analysis. For the solid tumor model, mice were subcutaneously inoculated with A549-luc cells (2.5×10⁶) and grouped similarly. Three days later, mice received MOCK, control, or FL-primed MSLN CAR-T cells (2×10⁶). Tumor burden was monitored using BLI and caliper measurements; body weight was tracked throughout. Peripheral blood was collected under inhalation anesthesia on day 12 post-treatment for T cell exhaustion and memory phenotype analysis. Mice were euthanized by gradual displacement of air with CO₂, with the flow rate adjusted to achieve complete air replacement within 2-3 minutes, followed by an additional 2-3-minute observation period to confirm death. To assess the exhaustion and memory phenotypes of tumor-infiltrating T cells, subcutaneous tumor xenografts were harvested on day 30 post-infusion, enzymatically dissociated into single-cell suspensions, and analyzed by flow cytometry.

### Histological Analysis

At the endpoint, tumor tissues (for CD3+ T cell infiltration) were collected for histological evaluation. Tissues were fixed in 10% neutral buffered formalin for 72 hours, processed using standard paraffin embedding, and sectioned at 5 μm thickness. For evaluation of T cell infiltration, the sections were stained with an anti-CD3 antibody (IHC; ZSGB-BIO), following the manufacturers' protocols. For histopathological assessment, sections were subjected to HE (Servicebio) staining following standard protocols. Images were acquired using an Olympus microscope.

### Bulk RNA Sequencing

Total RNA was extracted from control or FL-primed MSLN CAR-T cells using TRIzol (Invitrogen). RNA quality was assessed with Agilent 2200. Libraries were prepared using VAHTS Universal V6 RNA-seq Library Prep Kit (Vazyme) and sequenced on the DNBSEQ-T7 platform (2×150 bp paired-end). Reads were filtered and aligned to the human genome (GRCh38, Ensembl v104) using STAR 21. Gene counts were obtained using HTSeq, and expression levels were quantified by RPKM. Differential expression analysis was conducted using DESeq2 with thresholds |log₂(fold change)| > 0.585 and FDR < 0.05.

### Seahorse Metabolic Assays

Mitochondrial and glycolytic function were assessed using the Seahorse XF Cell Mito Stress Test and Glycolysis Stress Test (Agilent). CAR-T cells (1×10⁵) were seeded on poly-L-lysine-coated culture microplates in assay medium. OCR was measured at baseline and after sequential injections of oligomycin (2 μM), FCCP (2 μM), and rotenone and antimycin A (0.5 μM). ECAR was assessed after glucose (10 mM), oligomycin (2 μM), and 2-DG (50 mM). Data were analyzed with Wave software (Agilent).

### Metabolic Flux Analysis Using [U-¹³C₆] Glucose Tracing

Control and FL-primed CAR-T cells were resuspended in glucose-free RPMI-1640 medium supplemented with 11 mM [U-¹³C₆] glucose (MedChemExpress), 1% GlutaMAX (Gibco), and 5 ng/mL IL-2, and incubated at 37°C for 12 hours. Following incubation, cells were counted and harvested rapidly by centrifugation at 1400 rpm for 5 minutes. Cell pellets were immediately quenched in liquid nitrogen for 1 minute and stored at -80°C until submission to Biotree Biotech Co., Ltd. for liquid chromatography-mass spectrometry (LC-MS) analysis.

LC-MS data were processed to determine the isotopologue distributions and abundances of intracellular metabolites. XCalibur software was used to analyze mass isotopomer distributions (M+0, M+1, M+2, etc.), with corrections applied for natural ¹³C abundance. Labeling patterns of key glycolytic and tricarboxylic acid (TCA) cycle intermediates were quantified. Specifically, M+3 enrichment in lactate was used to assess glycolytic flux, while M+2 enrichment in citrate, isocitrate, α-ketoglutarate, succinate, fumarate, and malate was used as a surrogate for mitochondrial (TCA cycle) flux.

### Measurement of Lactate Production

The level of lactate production was measured by using the L-Lactate Assay Kit (Beyotime) following the manufacturer's instructions. Briefly, the control and FL-primed MSLN CAR-T cells (1×10⁶) were collected and washed with cold PBS buffer, lysed in 100 μL BeyoLysis™ Buffer A, centrifuged at 12,000 × g for 5 min at 4 °C, and the supernatants were used for lactate measurement. For each reaction, 50 μL of cell supernatant was added to a 96-well plate, followed by 50 μL of working solution (containing lactate assay buffer, enzyme solution, substrate, and WST-8 reagent). After incubation at 37 °C for 30 min in the dark, absorbance at 450 nm was measured using a microplate reader.

### Measurement of Intracellular ATP Level

The level of intracellular ATP was measured by using the ATP Assay Kit (Beyotime) following the manufacturer's instructions. Briefly, the control and FL-primed MSLN CAR-T cells (1×10⁶) were collected and washed with cold PBS buffer, lysed in 100 μL ATP lysis buffer, centrifuged at 12,000 × g for 5 min at 4 °C, and the supernatants were used for ATP measurement. ATP detection working solution was prepared by diluting the ATP reagent 1:9 with the supplied dilution buffer. A total of 100 μL of working solution was added to a 96-well plate, followed by 20 μL of sample. Luminescence was measured using a luminometer.

### Transmission Electron Microscopy (TEM)

A total of 3×10⁶ control or FL-primed MSLN CAR-T cells were harvested, washed with cold 1×PBS and immediately fixed in 2.5% glutaraldehyde in 0.1 M sodium cacodylate buffer (pH 7.4) for 2 hours at room temperature in the dark, followed by post-fixation with 1% osmium tetroxide for 1 hour. The samples were subsequently dehydrated through a graded ethanol series and embedded in epoxy resin. Ultrathin sections (70 nm) were obtained using an ultramicrotome (Leica EM UC7), and stained with 2% uranyl acetate for 20 min and lead citrate for 10 min. Representative images were captured using a transmission electron microscope (Hitachi TEM system HT7800) operated at an accelerating voltage of 80 keV.

### Western Blot

2×10^6^ CAR-T cells were harvested following indicated treatments, washed with ice-cold 1×PBS, and lysed on ice for 30 min in RIPA lysis buffer supplemented with protease inhibitor cocktail and phosphatase inhibitors. The lysates were centrifuged at 12,000 rpm for 10 min at 4 °C. The supernatants were collected, mixed with 5×loading buffer, and denatured at 100 °C for 10 min. Equal amounts of lysates were loaded onto SDS-PAGE gels for separation and then transferred to PVDF membranes. Membranes were blocked with 5% BSA in TBST, incubated with primary antibodies including p-ERK_1/2_ (CST, Cat# 4370, 1:1000), ERK_1/2_ (CST, Cat# 4695, 1:1000) and β-actin (CST, Cat# 3700, 1:4000) at 4 °C overnight, followed by incubation with HRP-conjugated secondary antibodies at room temperature for 1 h. Protein bands were visualized using an ECL detection system.

### Single-Cell RNA Sequencing Analysis

The scRNA-seq data were processed as described previously 22. Integration anchors were identified using Seurat's FindIntegrationAnchors with 1-25 dimensions and 3,000 variable features. Further processing included data scaling (ScaleData), dimensionality reduction (RunPCA), batch effect correction (Harmony), shared nearest neighbor graph construction (FindNeighbors), clustering (FindClusters) and cluster annotation (FindAllMarkers). CAR-T cells were stratified into HMGCR-high and -low groups, based on the median gene expression level of HMGCR, and subsequently, the exhaustion signature scores were calculated using AddModuleScore.

### Statistical Analysis

Data analysis was conducted using GraphPad Prism 9.5.0, FlowJo v10, Fiji, Adobe Illustrator 2020 and R studio (4.2.1). Results are presented as mean ± SEM from at least three independent experiments. Statistical comparisons were made using unpaired two-tailed Student's t-test, multiple t-tests with FDR correction (Benjamini-Hochberg), one-way or two-way ANOVA followed by Tukey's post hoc test, or log-rank test, as appropriate. Statistical significance was denoted as ns (not significant), *P < 0.05, **P < 0.01, ***P < 0.001.

## Results

### Cholesterol Accumulation During *Ex Vivo* Manufacturing Drives Baseline Exhaustion in CAR-T Cells

Chimeric antigen receptor (CAR)-T cells are typically generated through a 7-10-day *ex vivo* manufacturing period, during which T cells undergo activation, transduction, and expansion. This window constitutes the core phase for shaping the functional properties of the final cellular product. In our study, we focused on this manufacturing period, using day 7—the endpoint of *ex vivo* culture—as a representative timepoint to evaluate CAR-T cell phenotype.

**Figure 1:**
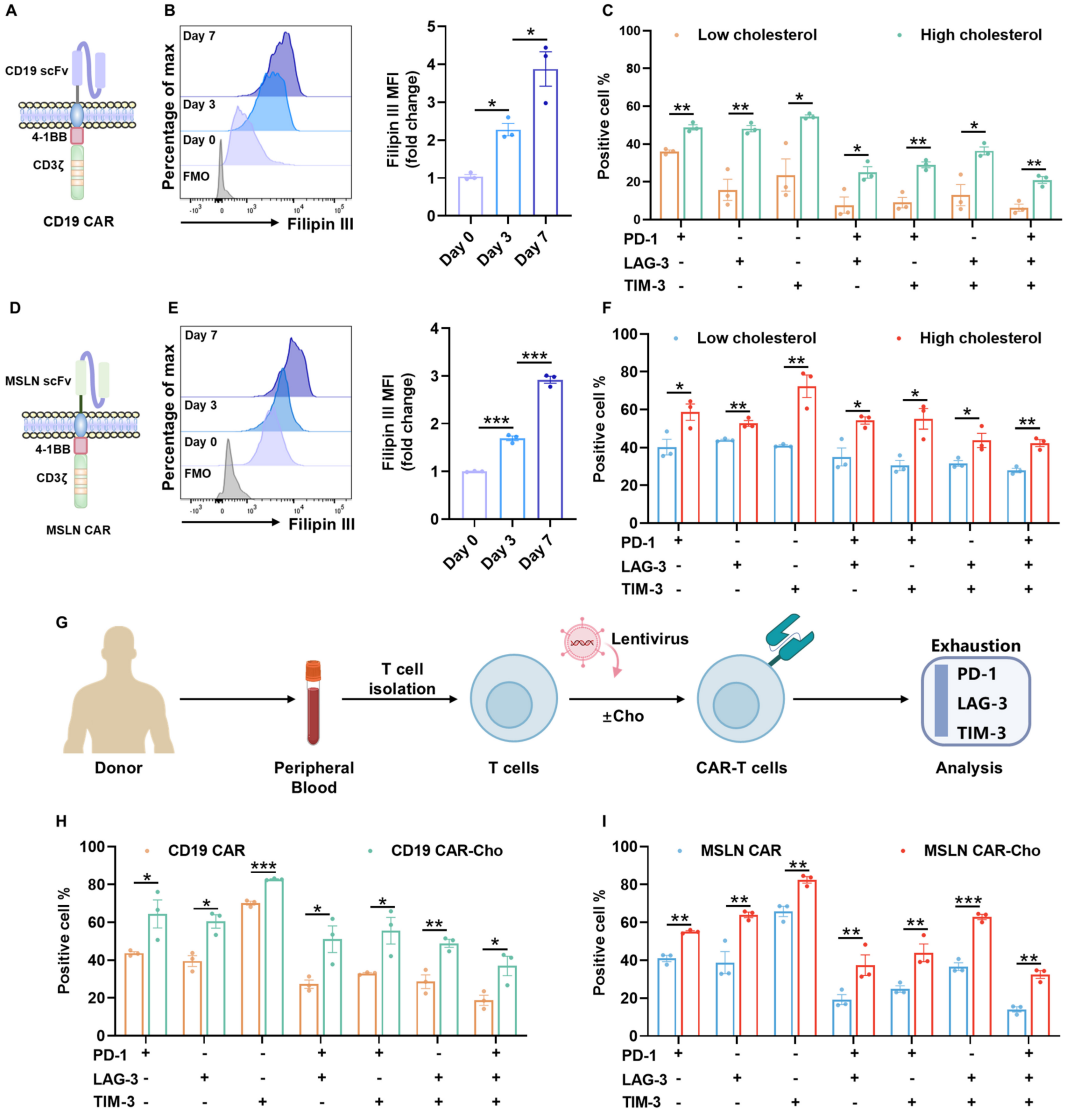
** Cholesterol accumulation during *ex vivo* biomanufacturing contributes to exhaustion in CAR-T cells. (A)** Schematic of the second-generation CD19 CAR construct comprising a CD28 hinge, 4-1BB costimulatory, and CD3ζ signaling domains. **(B)** Representative flow cytometry histogram and quantitative analysis of Filipin III staining showing dynamic cholesterol accumulation in CD19 CAR-T cells from day 0 to day 7 post-activation (n=3). **(C)** Co-expression of exhaustion-associated inhibitory receptors (PD-1, LAG-3, and TIM-3) in CD19 CAR-T cell subsets with low versus high intracellular cholesterol levels (n=3). **(D)** Schematic of the second-generation MSLN CAR construct comprising a CD8α hinge, 4-1BB costimulatory, and CD3ζ signaling domains. **(E)** Representative histogram and quantification of Filipin III staining in MSLN CAR-T cells from day 0 to day 7 post-activation (n=3). **(F)** Co-expression of PD-1, LAG-3, and TIM-3 in MSLN CAR-T cell subsets with low versus high cholesterol levels (n=3). **(G)** Experimental workflow for cholesterol supplementation. Peripheral blood T cells were isolated from different healthy donors and cultured in the presence or absence of exogenous cholesterol. Exhaustion phenotypes were assessed by flow cytometry. **(H-I)** Co-expression of PD-1, LAG-3, and TIM-3 in CD19 (H) and MSLN (I) CAR-T cells cultured with or without exogenous cholesterol supplementation (n=3). Data are presented as mean ± SEM from three independent experiments. Statistical significance was determined using multiple t-tests with false discovery rate (FDR) correction (Benjamini-Hochberg method) (C, F, H, and I), and one-way ANOVA followed by Tukey's post hoc test (B and E). Significance is indicated as *P < 0.05, **P < 0.01, ***P < 0.001.

To characterize cholesterol dynamics during CD19 CAR-T cell manufacturing, we monitored total cellular cholesterol levels from day 0 to day 7 following T cell activation (Figure 1A). Filipin III staining and flow cytometry revealed a progressive accumulation of cholesterol throughout the culture period (Figure 1B), consistent with active biosynthesis and uptake in rapidly expanding T cells 23, 24.

To assess the functional implications of elevated cholesterol, we reanalyzed a published single-cell RNA-sequencing dataset of peripheral blood CD19 CAR-T cells from treated patients 22. Cells with higher expression of HMGCR, the rate-limiting enzyme in cholesterol synthesis, showed enrichment of exhaustion-associated transcriptional programs (Figure S1A-C).

We further validated these findings by stratifying cultured CD19 CAR-T cells on day 7 based on the mean fluorescence intensity (MFI) of Filipin III staining. The median (50% MFI) value was used as the cut-off to define the high-cholesterol and low-cholesterol subgroups. Cells within the high-cholesterol subgroup exhibited markedly increased surface co-expression of exhaustion markers, including PD-1, LAG-3, and TIM-3, compared to their low-cholesterol counterparts (Figure 1C, Figure S2A). This cholesterol-exhaustion association was similarly observed in MSLN-targeted CAR-T cells (Figure 1D-F, Figure S2B), suggesting that the effect is not dependent on CAR specificity.

Moreover, supplementation of exogenous cholesterol during the culture process led to further cholesterol accumulation and exacerbated exhaustion phenotypes in both CD19 and MSLN CAR-T cells (Figure 1G-I, Figure S3A-B). Taken together, these results demonstrate that excessive cholesterol accumulation—whether endogenous or exogenously induced—during *ex vivo* expansion promotes CAR-T cell baseline exhaustion, potentially compromising their therapeutic efficacy.

### Low-dose FL Priming Enhances CAR-T Cell Phenotype and Function

**Figure 2:**
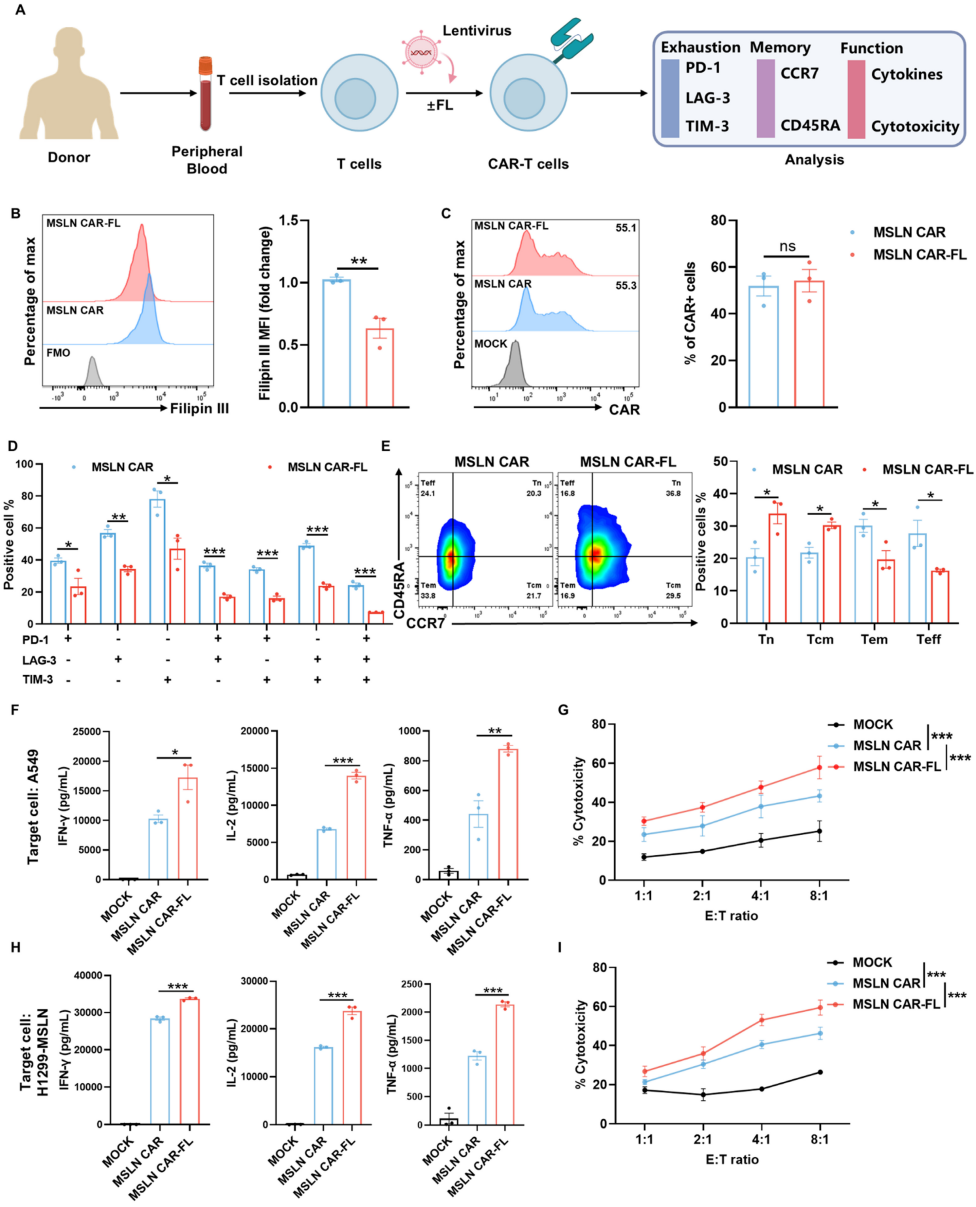
** Low-dose FL priming enhances phenotype and function of MSLN CAR-T cells. (A)** Experimental design of FL priming. Peripheral blood T cells were isolated from different healthy donors and cultured with or without FL supplementation. CAR-T cell exhaustion, memory phenotypes, and functions were evaluated by flow cytometry or ELISA. **(B)** Representative histogram and quantification of Filipin III staining showing reduced cholesterol levels in MSLN CAR-T cells with FL treatment (n=3). **(C)** MSLN CAR surface expression in the presence or absence of FL, as measured by flow cytometry (n=3). **(D-E)** Phenotypic effects of FL priming on MSLN CAR-T cells. (D) Co-expression of PD-1, LAG-3, and TIM-3; (E) Memory phenotype based on CCR7 and CD45RA expression (n=3). **(F)** ELISA quantification of IFN-γ, IL-2, and TNF-α levels in supernatants of control or FL-primed MSLN CAR-T cells after 24-hour co-culture with A549 cells at an E:T ratio of 2:1 (n=3). **(G)** Cytotoxicity of control or FL-primed MSLN CAR-T cells against A549 cells at indicated E:T ratios, assessed by CFSE/PI staining (n=4). **(H)** ELISA quantification of IFN-γ, IL-2, and TNF-α levels in supernatants of control or FL-primed MSLN CAR-T cells after 24-hour co-culture with H1299-MSLN cells at an E:T ratio of 2:1 (n=3). **(I)** Cytotoxicity of control or FL-primed MSLN CAR-T cells against H1299-MSLN cells at indicated E:T ratios, assessed by CFSE/PI staining (n=3). Data are presented as mean ± SEM from three independent experiments. Statistical analysis was performed using unpaired two-tailed Student's t-test (B, C), multiple t-tests with FDR correction (Benjamini-Hochberg method) (D, E), one-way ANOVA (F, H) or two-way ANOVA (G, I), followed by Tukey's post hoc test. *P < 0.05, **P < 0.01, ***P < 0.001.

Given the detrimental effect of cholesterol accumulation, we next tested whether restricting cholesterol biosynthesis during CAR-T cell manufacturing could improve the quality of the CAR-T cell product (Figure 2A). To define the working dose window of FL, we systematically evaluated a range of FL concentrations in both MSLN CAR-T cells and CD19 CAR-T cells, and assessed three matched readouts, including cell viability, cell proliferation, and cholesterol levels. The results showed that FL at 10 nM or lower had minimal effects on cell viability or proliferation, whereas further increases in FL concentration led to a dose-dependent reduction in both viability and proliferative capacity, suggesting that higher-dose FL priming may impair CAR-T cell expansion (Figure S4A-B). We then examined the total cholesterol levels and found that FL reduced cholesterol content in a dose-dependent manner. Notably, 10 nM FL was already sufficient to significantly decrease cholesterol levels in MSLN CAR-T cells and CD19 CAR-T cells, while exerting only minimal effects on viability and proliferation (Figure S4A-B). Therefore, based on the balance between cholesterol-lowering efficacy and maintenance of cell viability and proliferative capacity, we selected 10 nM FL as the working concentration for all subsequent functional and mechanistic studies. Given that cholesterol is an essential structural component of the plasma membrane and is required for T cell proliferation and TCR signaling, excessive suppression of cholesterol biosynthesis can impair T-cell growth 24, 25. This explains why higher FL doses that overly restrict cholesterol availability negatively affected CAR-T cell viability and proliferation.

At this optimized low dose, FL significantly lowered cellular cholesterol levels in MSLN CAR-T cells (Figure 2B) without altering CAR expression (Figure 2C). FL-primed MSLN CAR-T cells expressed lower levels of exhaustion markers, involving PD-1, LAG-3, and TIM-3 (Figure 2D, Figure S5), and displayed a shift toward a less-differentiated phenotype, with increased proportions of CD45RA+CCR7+ naïve (Tn) and CD45RA-CCR7+ central memory (Tcm) subsets (Figure 2E). These characteristics are closely associated with enhanced persistence and antitumor function. Accordingly, we performed additional analyses focusing on exhaustion- and memory-associated gene signatures. The results showed a consistent transcriptional shift toward a less exhausted and more memory-like state in FL-primed CAR-T cells, as reflected by reduced expression of multiple exhaustion-associated genes (e.g., LAG3, HAVCR2, and PRDM1) and increased expression of memory-related genes (e.g., TCF7, LEF1, IL7R, and BACH2), as well as genes associated with exhaustion resistance (e.g., CX3CR1 and JUN) (Figure S6). While not all individual exhaustion markers showed uniform changes at the mRNA level, the overall transcriptional profile is consistent with the phenotypic data, supporting reduced exhaustion and enhanced memory formation in FL-primed CAR-T cells. Similar results were observed in CD19 CAR-T cells treated with 10 nM FL, including reduced cholesterol levels (Figure S7A), unaltered CAR expression (Figure S7B), decreased exhaustion levels (Figure S7C, Figure S8), and increased proportions of Tn and Tcm subsets (Figure S7D). These findings indicate that low-dose FL priming could broadly improve CAR-T cell phenotype across different CAR constructs and antigen targets.

We next assessed whether the improved phenotype conferred by FL priming translated into enhanced effector function. Upon stimulation with targeted tumor cells, FL-primed MSLN CAR-T cells secreted significantly higher levels of key effector cytokines, including IFN-γ, IL-2, and TNF-α, compared to control CAR-T cells (Figure 2F, H, Figure S9). In co-culture cytotoxicity assays, FL-primed CAR-T cells also exhibited greater tumor cell killing ability across multiple E:T ratios (Figure 2G, I). Similar results were observed in CD19 CAR-T cells treated with 10 nM FL (Figure S7E-H).

**Figure 3:**
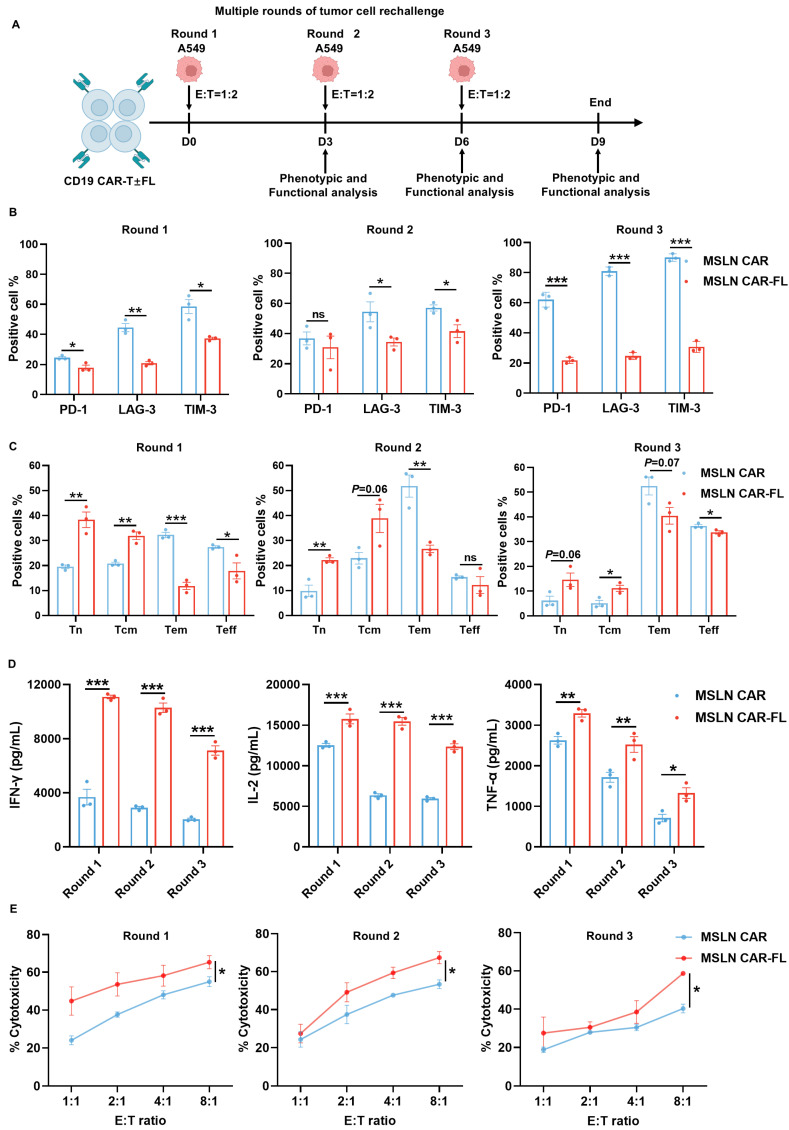
**FL priming enhances the persistence of MSLN CAR-T cell antitumor activity upon serial tumor cell rechallenge. (A)** Schematic diagram of the serial tumor cell rechallenge experimental design. MSLN CAR-T cells with or without FL priming were subjected to multiple rounds of targeted tumor cell rechallenge at an E:T ratio of 1:2 on day 0, 3, and 6, followed by phenotypic and functional analysis after each round of rechallenge on day 3, 6, and 9. **(B-C)** Phenotypic effects of FL priming on MSLN CAR-T cells upon serial tumor cell rechallenge. (B) The expression of PD-1, LAG-3, and TIM-3; (C) Memory phenotype based on CCR7 and CD45RA expression (n=3). **(D)** Cytokine secretion of MSLN CAR-T cells after each round of rechallenge. Concentrations of IFN-γ, IL-2, and TNF-α in culture supernatants were measured by ELISA (n=3). **(E)** Serial cytotoxicity assays of MSLN CAR-T cells against A549. The cytotoxicity of MSLN CAR-T cells was assessed at indicated E:T ratios after each round of rechallenge, measured by CFSE/PI staining (n=3). Data are shown as mean ± SEM. Statistical significance was determined by multiple t-tests with FDR correction (Benjamini-Hochberg method) (B, C, D), or two-way ANOVA with multiple comparisons (E). *P < 0.05, **P < 0.01, ***P < 0.001.

Chronic antigen exposure induces CAR-T cell exhaustion, which compromises their sustained antitumor activity 26-28. Since FL priming has been shown to mitigate exhaustion and augment CAR-T cell persistence, we subsequently investigated whether similar benefits of FL priming extend to chronic antigen stimulation (Figure 3A). The results showed that FL-primed MSLN CAR-T cells expressed lower levels of exhaustion markers, involving PD-1, LAG-3, and TIM-3 (Figure 3B), and displayed a shift toward a less-differentiated Tn and Tcm phenotype (Figure 3C). As expected, FL-primed MSLN CAR-T cells exhibited significantly elevated secretion of cytokines and superior tumor cell killing ability, compared with control MSLN CAR-T cells across multiple rounds of antigen rechallenge (Figure 3D-E). Consistent results were observed with FL-primed CD19 CAR-T cells (Figure S10). Collectively, these findings indicate that FL priming not only mitigates CAR-T cell exhaustion and promotes the formation of memory but also augments the persistence of CAR-T cell effector functions during both acute and chronic antigen stimulation *in vitro.*

### Low-Dose FL Priming Improves *In Vivo* Persistence and Antitumor Efficacy of CAR-T Cells

**Figure 4:**
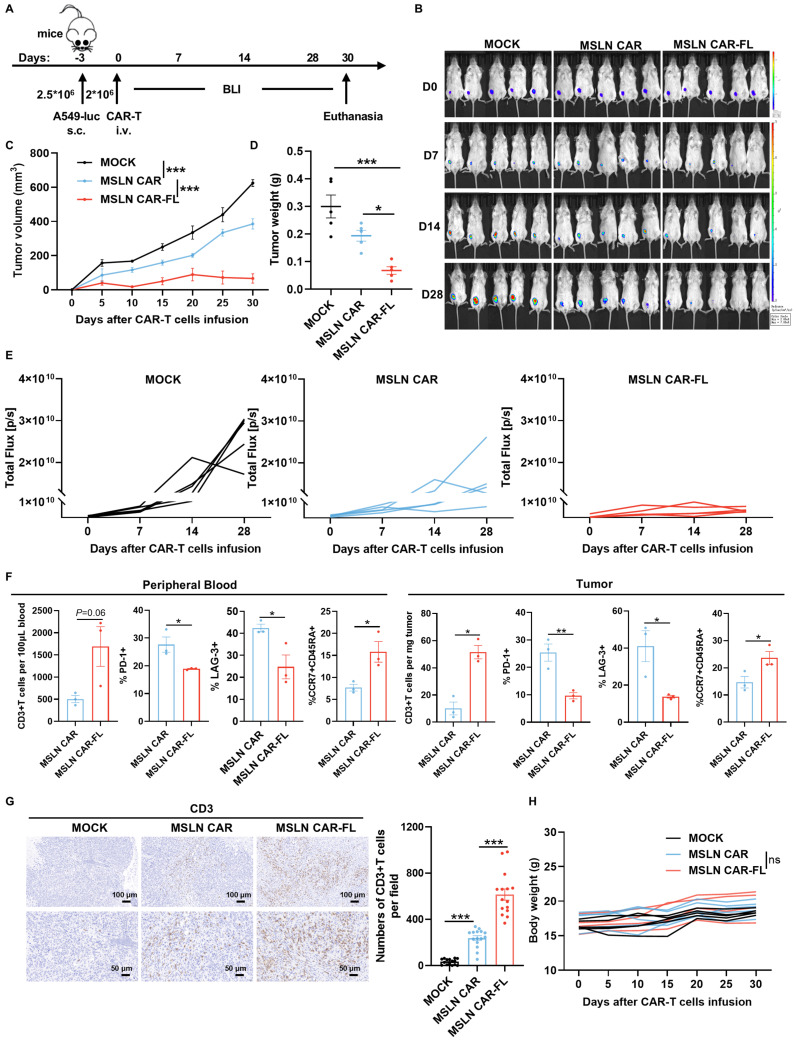
** FL priming augments the antitumor activity of MSLN CAR-T cells in a subcutaneous tumor model. (A)** Experimental design: NCG mice were subcutaneously injected with A549-luc cells (2.5×10⁶) and treated with MOCK, control, or FL-primed MSLN CAR-T cells (2×10⁶) on day 0. **(B)** Tumor burden monitored by BLI imaging (n=5). **(C)** The tumor volume curves from caliper measurements (n=5). **(D)** The tumor weights at endpoint (n=5). **(E)** Quantification of BLI signals (n=5). **(F)** Flow cytometry of peripheral blood and tumor-infiltrating T cells: CD3+ cell counts, exhaustion markers (PD-1, LAG-3) and naïve subset (CCR7+CD45RA+) on day 12 and day 30, respectively (n=3). **(G)** CD3+ T cell infiltration in tumors on day 30 assessed by IHC (left: representative images; right: quantification). **(H)** Longitudinal body weight measurements (n=5). Statistical significance was determined using two-way ANOVA with Tukey's post hoc test (C, H), one-way ANOVA with Tukey's post hoc test (D, G) and unpaired two-tailed Student's t-test (F). *P < 0.05, **P < 0.01, ***P < 0.001; ns, not significant.

To determine whether FL priming enhances CAR-T cell therapeutic efficacy *in vivo*, we evaluated its effect in both solid tumor and hematologic xenograft models. In the subcutaneous A549-luc lung cancer model, mice treated with FL-primed MSLN CAR-T cells exhibited superior tumor growth control compared to those receiving control MSLN CAR-T cells or MOCK T cells (Figure 4A-E). Flow cytometric analysis of peripheral blood and tumor-infiltrating lymphocytes revealed an increased abundance of CD3+ T cells, accompanied by reduced expression of exhaustion markers and an enhanced memory phenotype in the FL-primed group (Figure 4F). Immunohistochemistry further confirmed significantly greater intratumoral CD3+ T cell infiltration in mice treated with FL-primed CAR-T cells (Figure 4G). Importantly, FL-primed CAR-T cells did not induce any notable mouse body weight loss (Figure 4H). These findings confirm that FL priming improves the* in vivo* antitumor activity of CAR-T cells in solid tumor settings. Moreover, we have also assessed representative indicators of liver and kidney function, including ALT, AST, TBIL, DBIL, ALP, γ-GT, BUN, and UA. No statistically significant differences were detected among the three groups (Figure S11A-H). HE staining of major organs, including the heart, liver, spleen, lung, and kidney, did not reveal obvious pathological abnormalities or treatment-related tissue damage in either CAR-T-treated group (Figure S11I).

Similarly, in the Raji-luciferase lymphoma model, mice that received FL-primed CD19 CAR-T cells exhibited more rapid tumor regression and prolonged survival compared to those receiving control CD19 CAR-T cells (Figure S12A-D). Longitudinal analysis revealed that FL-primed CD19 CAR-T cells exhibited superior persistence in peripheral blood, expressed lower levels of exhaustion markers and maintained higher proportions of Tn subsets (Figure S12E). No significant weight loss was observed (Figure S12F).

### FL Priming Reprograms CAR-T Cell Metabolism Toward OXPHOS in an ERK_1/2_-Dependent Manner

**Figure 5:**
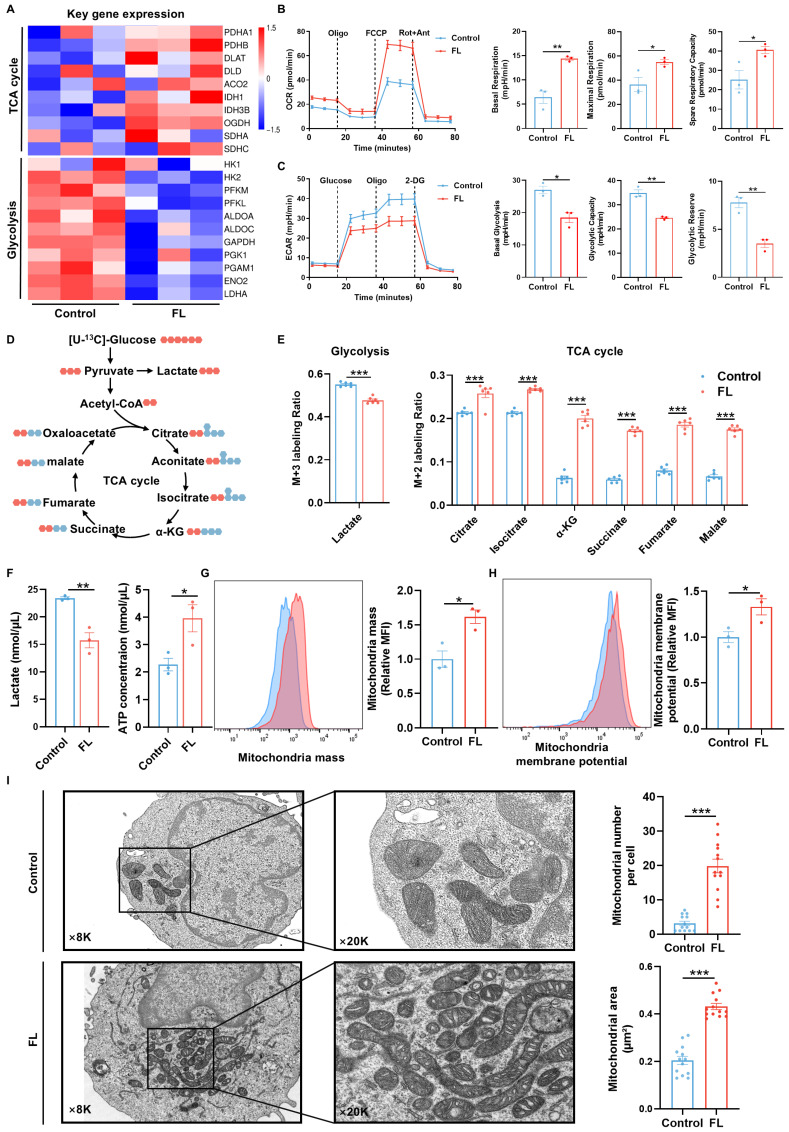
** FL priming remodels CAR-T cell metabolism to promote functional fitness. (A)** Heatmap of TCA cycle- and glycolysis-related gene expression profiles (n=3). **(B)** OCR curves of control and FL-primed MSLN CAR-T cells measured under mitochondrial stress test; quantification of basal respiration, maximal respiration, and spare respiratory capacity (n=3). **(C)** ECAR curves and quantification of glycolysis parameters: basal glycolysis, glycolytic capacity, and glycolytic reserve (n=3). **(D)** Schematic representation of [U-¹³C_6_]-glucose carbon tracing patterns. **(E)** Labeling ratio of intermediate metabolites in glycolysis and TCA cycle derived from [U-¹³C_6_]-glucose. Lactate (M+3), citrate (M+2), isocitrate (M+2), α-KG (M+2), succinate (M+2), fumarate (M+2) and malate (M+2) were shown (n=6). **(F)** Lactate production and ATP levels of control and FL-primed MSLN CAR-T cells (n=3). **(G-H)** Representative flow cytometry plots and quantification of mitochondrial mass (G) and mitochondrial membrane potential (H) (n=3). **(I)** The mitochondrial ultrastructure of control and FL-primed MSLN CAR-T cells. Left: representative electron microscopy images. Right: quantification analysis of mitochondrial number, mitochondrial area (n=13). Data are presented as mean ± SEM. Statistical tests included unpaired two-tailed Student's t-test (B, C, E, F, G, H and I). *P < 0.05, **P < 0.01, ***P < 0.001.

To elucidate the mechanisms underlying the improved functionality of FL-primed CAR-T cells, we performed bulk RNA sequencing of MSLN CAR-T cells cultured in the presence or absence of FL. Transcriptomic analysis revealed a downregulation of glycolytic genes and an upregulation of TCA cycle-related pathways in FL-primed cells (Figure 5A), suggesting a metabolic shift toward OXPHOS.

This metabolic shift was further validated by Seahorse analysis, which showed that FL-primed CAR-T cells exhibited elevated oxygen consumption rates (OCR), increased basal and maximal respiration and spare respiratory capacity (SRC), along with reduced extracellular acidification rates (ECAR), glycolytic capacity and glycolytic reserve (Figure 5B-C). Moreover, FL-primed MSLN CAR-T cells showed a decreased glycolytic proton efflux rate (glycoPER), further supporting the inhibited glycolytic activity and lactate release induced by FL priming (Figure S13). These results were consistent with enhanced mitochondrial respiration and reduced glycolytic activity.

To further delineate metabolic flux, [U-¹³C₆] glucose tracing was performed. Compared to the control CAR-T cells, FL-primed CAR-T cells displayed reduced ¹³C incorporation into lactate—the end product of glycolysis—and increased ¹³C enrichment in multiple TCA cycle intermediates, including citrate, isocitrate, α-ketoglutarate (α-KG), succinate, fumarate, and malate (Figure 5D-E). These data suggest that FL priming suppresses glycolysis-derived lactate production by redirecting pyruvate flux into mitochondrial oxidative metabolism, thereby enhancing TCA cycle activity and OXPHOS. This result was further substantiated by lower lactate production and higher ATP levels observed in FL-primed MSLN CAR-T cells (Figure 5F).

Consistent with this metabolic reprogramming, FL-primed CAR-T cells showed increased mitochondrial mass and membrane potential as assessed by MitoTracker Green and MitoProbe^TM^ TMRM staining (Figure 5G-H). Additionally, transmission electron microscopy revealed a significant increase in mitochondrial mass and mitochondrial area in FL-primed CAR-T cells, providing further evidence of augmented mitochondrial biogenesis and function (Figure 5I).

**Figure 6:**
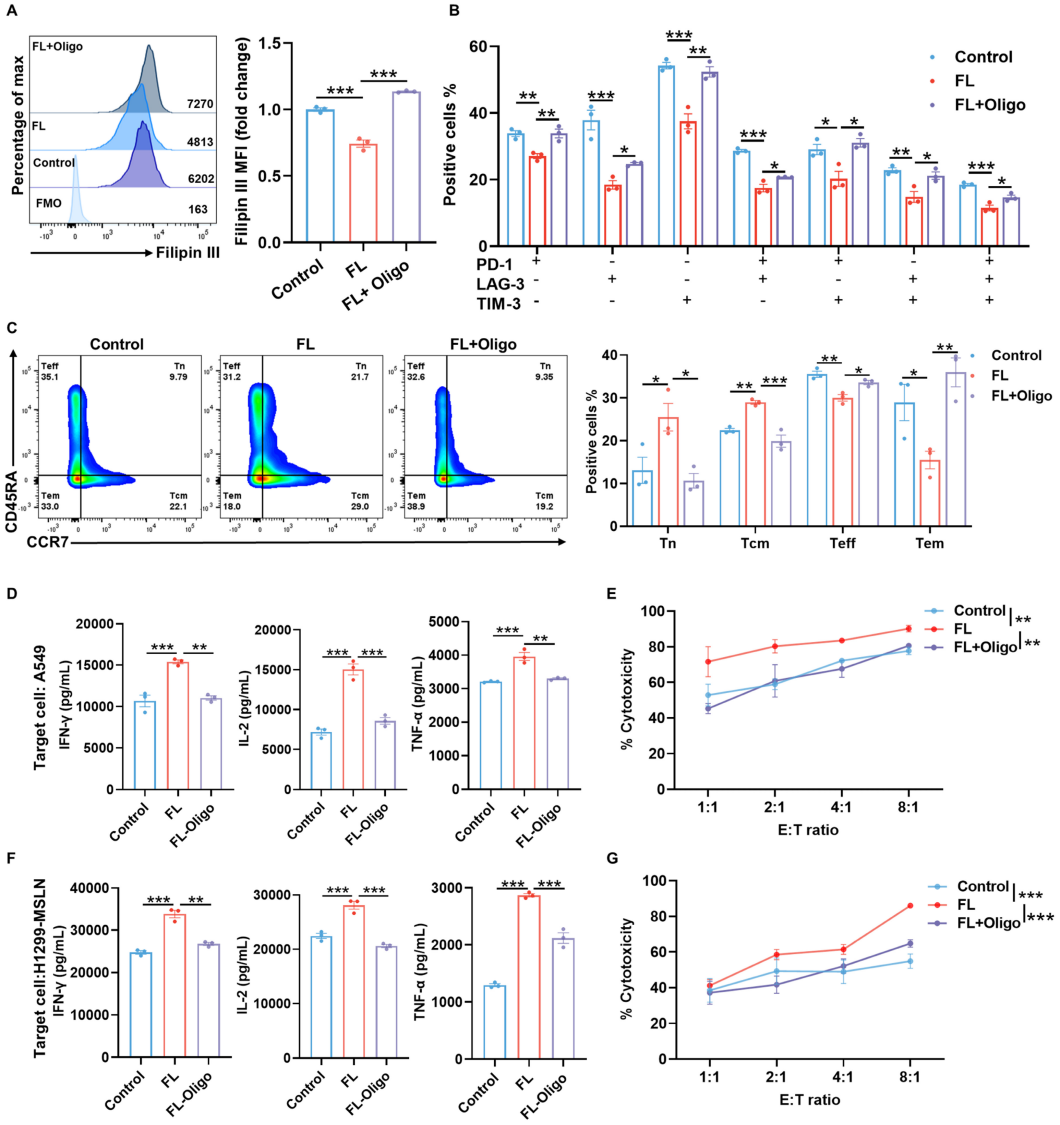
** The enhanced phenotypes and antitumor effects are abrogated by oligomycin treatment. (A)** Filipin III staining showing intracellular cholesterol levels in MSLN CAR-T cells under the indicated conditions. Cells were treated with oligomycin (Oligo, 2.5 μM), a mitochondrial ATP synthase inhibitor, for 3 days and analyzed by flow cytometry (n=3). **(B)** Expression of PD-1, LAG-3, and TIM-3 in MSLN CAR-T cells under indicated conditions (n=3). **(C)** Distribution of memory T cell subsets in MSLN CAR-T cells under indicated conditions (n=3). **(D)** Levels of IFN-γ, IL-2, and TNF-α in the supernatants of control MSLN CAR-T cells, FL-primed MSLN CAR-T cells, or FL-primed MSLN CAR-T cells treated with 2.5 μM oligomycin for 3 days after 24-hour co-culture with A549 cells at an E:T ratio of 2:1, measured by ELISA (n=3). **(E)** Cytotoxicity of control MSLN CAR-T cells, FL-primed MSLN CAR-T cells or FL-primed MSLN CAR-T cells treated with 2.5 μM oligomycin for 3 days against A549 cells at indicated E:T ratios, assessed by CFSE/PI staining (n=4). **(F)** Levels of IFN-γ, IL-2, and TNF-α in the supernatants of control MSLN CAR-T cells, FL-primed MSLN CAR-T cells, or FL-primed MSLN CAR-T cells treated with 2.5 μM oligomycin for 3 days after 24-hour co-culture with H1299-MSLN cells at an E:T ratio of 2:1, measured by ELISA (n=3). **(G)** Cytotoxicity of control MSLN CAR-T cells, FL-primed MSLN CAR-T cells or FL-primed MSLN CAR-T cells treated with 2.5 μM oligomycin for 3 days against H1299-MSLN cells at indicated E:T ratios, assessed by CFSE/PI staining (n=3). Data are presented as mean ± SEM from three independent experiments. Statistical significance was determined using one-way ANOVA (A, B, C, D, F) or two-way ANOVA (E, G), with Tukey's post hoc test. *P < 0.05, **P < 0.01, ***P < 0.001.

To determine whether this metabolic rewiring is functionally required for the observed phenotypic improvements, we treated CAR-T cells with 2.5 μM oligomycin, a mitochondrial ATP synthase inhibitor for 3 days. The results demonstrated that FL markedly reduced the cholesterol levels, while mitochondrial inhibition with oligomycin treatment largely reversed the cholesterol-lowering effect of FL, restoring cholesterol levels to those observed in control CAR-T cells (Figure 6A). Previous studies have shown that mitochondrial dysfunction can alter cholesterol metabolism and promote cellular cholesterol accumulation. Chronic mitochondrial dysfunction has been demonstrated to increase cellular cholesterol levels by upregulating the glutamine consumption and altering mevalonate pathway activation 29-31. Our observation that impaired OXPHOS is associated with cholesterol accumulation is consistent with these previous reports. Furthermore, oligomycin treatment abolished the beneficial effects of FL priming on exhaustion and memory phenotypes (Figure 6B-C, Figure S14). Moreover, FL-induced enhancements in cytokine production and cytotoxic activity were also reversed upon oligomycin treatment (Figure 6D-G), indicating that mitochondrial oxidative metabolism is indispensable for the improved functionality of FL-primed CAR-T cells.

**Figure 7:**
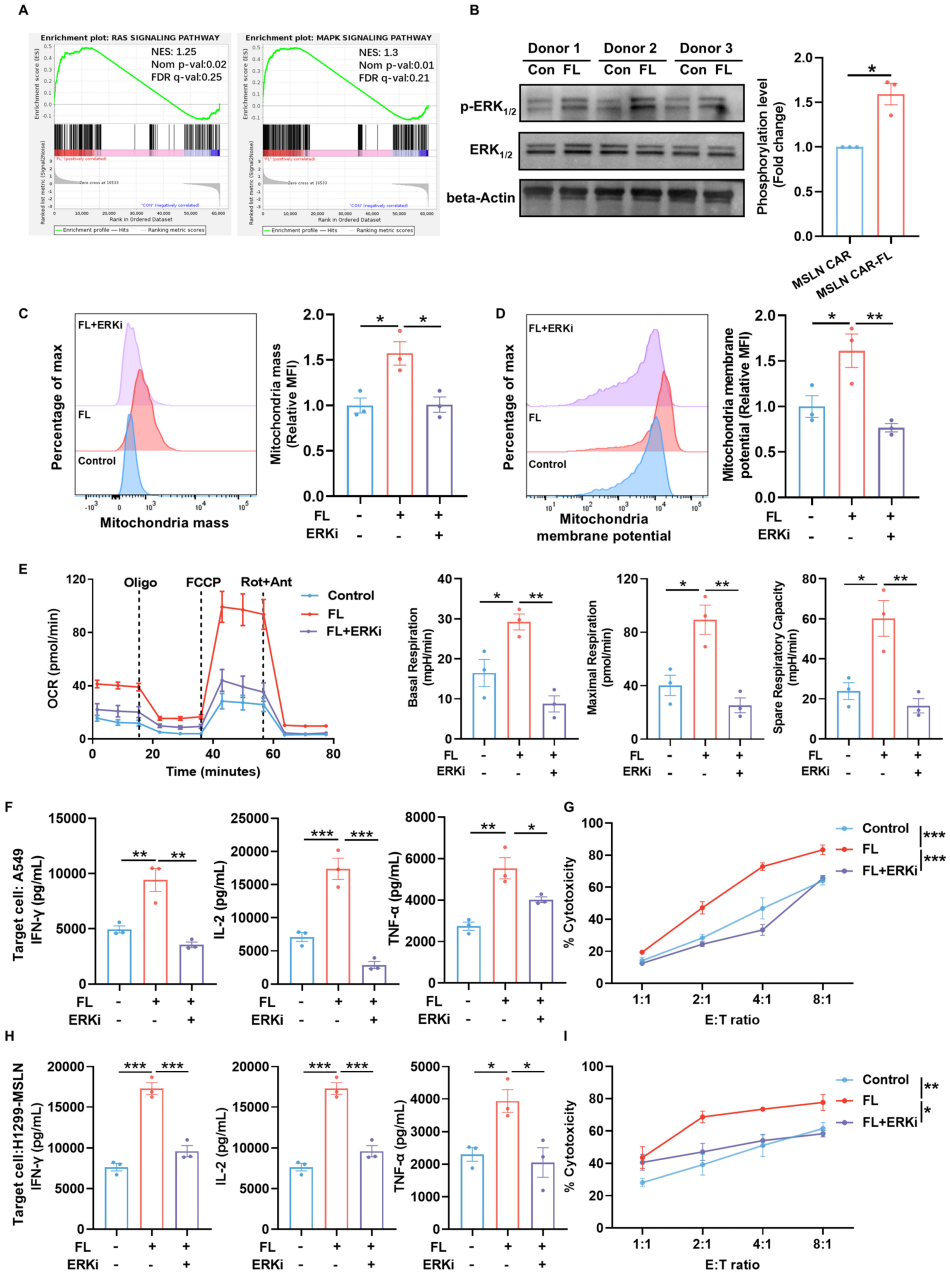
** FL enhances ERK_1/2_ phosphorylation to remodel CAR-T cell metabolism. (A)** GSEA showing enrichment of the RAS and MAPK signaling pathway in FL-primed CAR-T cells. **(B)** Representative immunoblots and quantification of p-ERK_1/2_, total ERK_1/2_, and β-actin in CAR-T cells from three donors (n=3). **(C-D)** Representative flow cytometry plots and quantification of mitochondrial mass (C) and mitochondrial membrane potential (D) in CAR-T cells treated with control, FL, or FL plus a selective ERK_1/2_ inhibitor (ERKi), SCH772984 (n=3). **(E)** OCR curves of control, FL-primed, and FL-primed plus ERKi MSLN CAR-T cells measured under mitochondrial stress test; quantification of basal respiration, maximal respiration, and spare respiratory capacity (n=3). **(F)** Levels of IFN-γ, IL-2, and TNF-α in the supernatants of control MSLN CAR-T cells, FL-primed MSLN CAR-T cells, or FL-primed MSLN CAR-T cells treated with ERKi for 3 days after 24-hour co-culture with A549 cells at an E:T ratio of 2:1, measured by ELISA (n=3). **(G)** Cytotoxicity of control MSLN CAR-T cells, FL-primed MSLN CAR-T cells or FL-primed MSLN CAR-T cells treated with ERKi for 3 days against A549 cells at indicated E:T ratios, assessed by CFSE/PI staining (n=3). **(H)** Levels of IFN-γ, IL-2, and TNF-α in the supernatants of control MSLN CAR-T cells, FL-primed MSLN CAR-T cells, or FL-primed MSLN CAR-T cells treated with ERKi for 3 days after 24-hour co-culture with H1299-MSLN cells at an E:T ratio of 2:1, measured by ELISA (n=3). **(I)** Cytotoxicity of control MSLN CAR-T cells, FL-primed MSLN CAR-T cells or FL-primed MSLN CAR-T cells treated with ERKi for 3 days against H1299-MSLN cells at indicated E:T ratios, assessed by CFSE/PI staining (n=3). Data are presented as mean ± SEM from three independent experiments. Statistical significance was determined using unpaired two-tailed Student's t-test (B), one-way ANOVA (C, D, E, F, H) or two-way ANOVA (G, I), with Tukey's post hoc test. *P < 0.05, **P < 0.01, ***P < 0.001.

To elucidate the mechanisms underlying the functional improvements induced by FL priming, we first performed gene set enrichment analysis (GSEA). This analysis revealed an enrichment of RAS/MAPK signaling pathway in FL-primed CAR-T cells, indicating engagement of MAPK-associated signaling programs (Figure 7A). Given that ERK_1/2_ is a central downstream effector of the RAS/MAPK cascade and a known regulator of T cell metabolism, we next examined ERK_1/2_ activation 32. Immunoblotting confirmed that FL priming markedly enhanced ERK_1/2_ phosphorylation across multiple donors (Figure 7B). This observation is consistent with previous studies demonstrating that moderate modulation of membrane cholesterol can alter lipid raft organization, thereby facilitating proximal TCR signaling events, including Lck and LAT recruitment, and enhancing the phosphorylation of ERK_1/2_^33^, ^34^. Given the established relationship between ERK_1/2_ signaling and T cell mitochondrial metabolism, we next assessed whether ERK_1/2_ phosphorylation was required for CAR-T cell metabolic and functional improvements conferred by FL priming 35, 36. We next treated CAR-T cells with 1 μM SCH772984, a selective ERK_1/2_ inhibitor for 3 days. Flow cytometric analysis demonstrated that the increased mitochondrial mass and elevated mitochondrial membrane potential induced by FL priming were reversed by the inhibition of ERK_1/2_ signaling (Figure 7C-D). Consistent with this observation, inhibiting ERK_1/2_ signaling largely abrogated the enhancement of mitochondrial respiration conferred by FL priming (Figure 7E). These findings demonstrated that FL-induced mitochondrial remodeling is ERK_1/2_ dependent. To determine whether ERK_1/2_-dependent metabolic remodeling translates into functional improvements, we evaluated cytokine production and cytotoxicity upon antigen stimulation. FL-primed MSLN CAR-T cells produced higher levels of effector cytokines relative to the control CAR-T cells, whereas ERK inhibition significantly diminished these increases (Figure 7F, H). Moreover, the enhanced CAR-T cell cytotoxicity across various E:T ratios was largely reversed by ERK_1/2_ blockade (Figure 7G, I). Collectively, these results indicate that FL priming enhances CAR-T cell mitochondrial fitness and antitumor function in an ERK_1/2_-dependent manner, and that ERK_1/2_-driven mitochondrial metabolism is essential for the functional benefits conferred by FL priming.

## Discussion

The therapeutic efficacy of CAR-T cell therapy is closely linked to the functional quality of the infused product. Clinical evidence has shown that CAR-T cells enriched in less differentiated, minimally exhausted subsets display superior expansion, persistence, and antitumor activity *in vivo* 12, 37. Therefore, optimizing cellular fitness during the manufacturing process is critical for improving therapeutic outcomes.

Our study identifies cellular cholesterol accumulation during *ex vivo* expansion as a previously underappreciated driver of T cell exhaustion in CAR-T cell manufacturing. We demonstrate that transient exposure to low-dose FL, a clinically approved HMG-CoA reductase inhibitor, can favorably reprogram CAR-T cells toward a memory-like, functionally potent state without impairing viability or proliferation. This intervention provides a simple, scalable strategy to improve CAR-T cell product quality.

Cholesterol plays a complex, context-dependent role in T cell biology. While sufficient cholesterol is essential for membrane biosynthesis, immune synapse formation, and TCR signaling, excessive accumulation—particularly in persistently stimulated or expanded T cells—can induce ER stress, disrupt membrane architecture, and promote terminal exhaustion 18, 24, 38. Conversely, cholesterol depletion below physiological thresholds impairs T cell proliferation and function 25, 39. These dual roles underscore the importance of maintaining cholesterol homeostasis within an optimal range to preserve T cell fitness.

To address this, we carefully titrated FL dosage and identified a very low concentration that attenuates cholesterol levels without compromising CAR-T cell expansion across multiple donors. FL-primed cells exhibited reduced expression of exhaustion markers (PD-1, LAG-3 and TIM-3), increased percentages of naïve and central memory subsets, and enhanced cytotoxicity* in vitro* and tumor control *in vivo*. Notably, these phenotypic improvements were accompanied by metabolic reprogramming toward oxidative phosphorylation—a hallmark of long-term memory T cells.

Although prior studies have explored the use of FL or other statins in modulating T cell function, these were typically conducted *in vivo*, with systemic drug administration affecting multiple cell types and immune compartments 40-42. Our study is distinct in its focus on CAR-T cells during their *ex vivo* expansion phase, a unique and controllable window in which T cell phenotype and metabolism can be precisely modulated. To our knowledge, this is the first report demonstrating that metabolic intervention with statins during CAR-T cell manufacturing improves the functional attributes of the final cellular product.

Compared to genetic engineering strategies—such as transcription factor overexpression (e.g., c-Jun, TCF1)—our approach is readily translatable. FL is FDA-approved, inexpensive, and compatible with GMP-compliant workflows. Its transient application simplifies regulatory considerations and enables straightforward combination with other enhancement strategies, such as cytokine supplementation, co-stimulatory tuning, or gene editing.

Mechanistically, our findings suggest that excessive cholesterol accumulation during *ex vivo* expansion contributes to CAR-T cell baseline exhaustion. Low-dose FL priming restored the cholesterol levels of CAR-T cells to a more physiological range, thereby remodeling the CAR-T cell metabolism and promoting a metabolic shift toward OXPHOS and a concomitant reduction in glycolytic activity. This is consistent with prior studies demonstrating that metabolic shifts toward OXPHOS boost the formation of less differentiated memory-like CAR-T cells with enhanced durability, whereas chronic antigen stimulation could drive CAR-T cells toward glycolysis, mitochondrial dysfunction, and exhaustion 9, 43-45. This metabolic reprogramming was confirmed by [U-¹³C₆] glucose tracing, Seahorse analysis, and transcriptomic profiling, all of which revealed enhanced mitochondrial respiration and biosynthetic pathways. Importantly, the beneficial effects of FL priming were largely abrogated by pharmacological inhibition of ERK_1/2_ signaling or mitochondrial ATP synthesis, supporting a critical role of ERK_1/2_-dependent mitochondrial metabolism in mediating the improved CAR-T cell phenotype and function. Together, these results highlight cholesterol homeostasis as a key and tunable regulator of CAR-T cell fate, acting in part through its impact on cellular metabolic fitness during manufacturing.

Nevertheless, three limitations merit discussion. First, while FL is well known to reduce cellular cholesterol levels, our current data do not definitively prove that cholesterol reduction is the sole driver of the observed metabolic reprogramming. Instead, our findings support a model in which cholesterol modulation is associated with downstream signaling and metabolic changes. Importantly, our data suggest that FL priming is associated with activation of the RAS/MAPK pathway and increased ERK_1/2_ phosphorylation. Moreover, ERK_1/2_ inhibitor rescue experiments demonstrated that blockade of ERK_1/2_ signaling attenuated the FL-mediated enhancement of mitochondrial oxidative metabolism and antitumor function, supporting that ERK_1/2_ activation is functionally involved in this process. Given prior evidence that membrane cholesterol can regulate lipid raft organization and downstream signaling, including ERK_1/2_ activation, and that ERK_1/2_ signaling is closely linked to mitochondrial metabolism, these findings suggest a potential cholesterol-ERK_1/2_-mitochondrial axis underlying the observed effects 33-36. Second, the early-intervention animal model does not fully recapitulate the clinical scenario, where tumors are typically more established and embedded within a more complex immunosuppressive microenvironment. Future studies using more advanced tumor models will be important to further validate the translational potential of this strategy. Finally, as FL targets the cholesterol biosynthetic pathway, additional cholesterol-associated processes, such as protein prenylation and membrane lipid raft organization, may also contribute to the observed phenotypic and functional changes in CAR-T cells. Further studies will be needed to dissect the relative contribution of these mechanisms.

**Figure 8:**
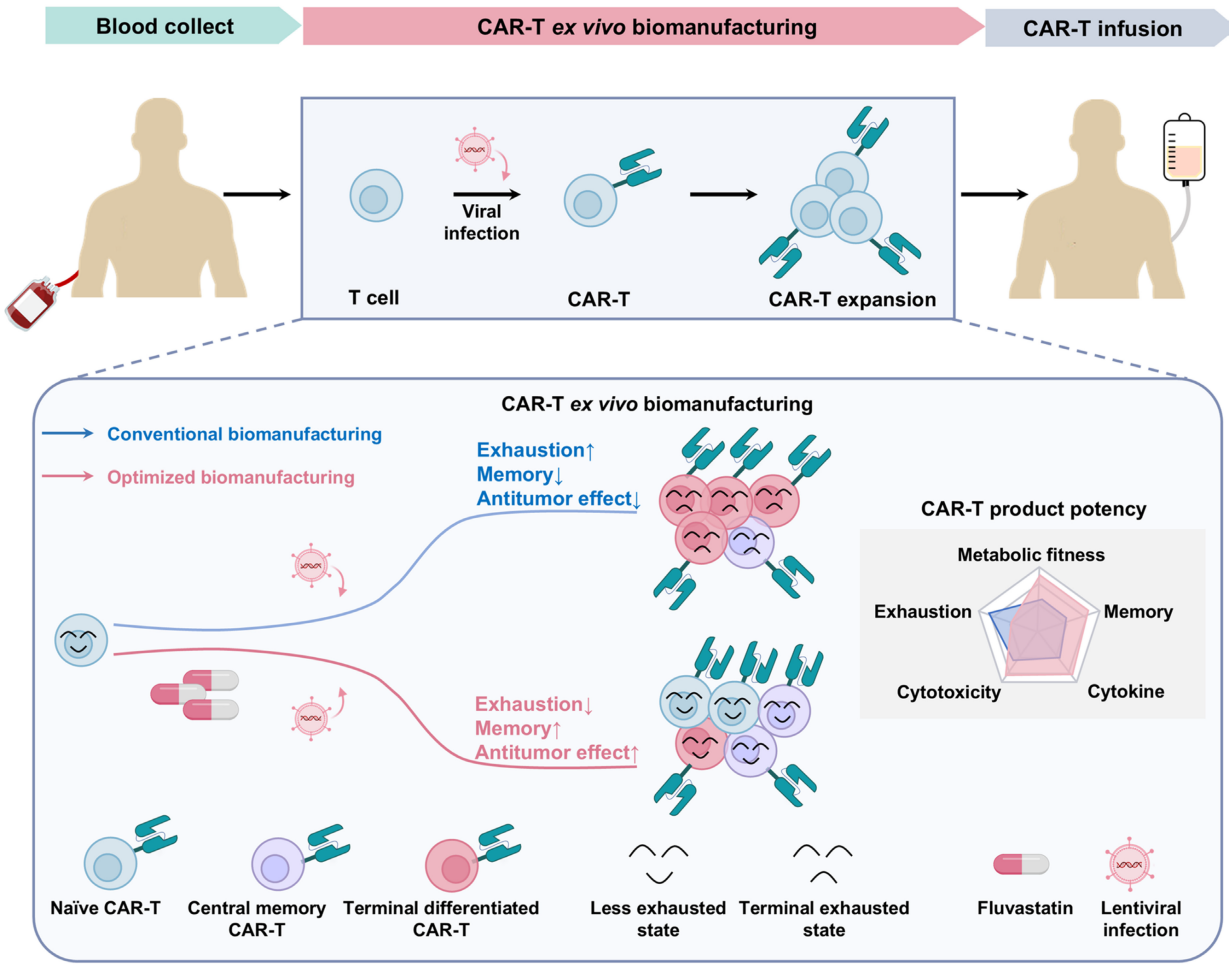
** Schematic summary of FL priming benefits on CAR-T cell fitness and function.** Low-dose FL priming during *ex vivo* CAR-T cell manufacturing reduces total cellular cholesterol accumulation, leading to a less exhausted, less differentiated, and memory-enriched T cell phenotype. This safe and practical *ex vivo* strategy enhances CAR-T cell efficacy in both hematologic malignancies and solid tumors.

In summary, our findings reveal that transient, low-dose FL treatment during *ex vivo* CAR-T cell expansion enhances the quality and therapeutic potential of the final cell product by reducing exhaustion and promoting memory formation (Figure 8). This work highlights cholesterol metabolism as a modifiable axis for improving CAR-T cell manufacturing and offers a clinically feasible approach to enhance the durability and efficacy of adoptive T cell therapies.

## Conclusion

Our study demonstrated that cholesterol accumulation during* ex vivo* manufacturing drives CAR-T cell exhaustion. By transiently modulating cholesterol biosynthesis with low-dose FL, we preserved a less-differentiated, memory-enriched phenotype, attenuated exhaustion, and enhanced CAR-T cell cytotoxicity, metabolic fitness, and *in vivo* persistence, without compromising viability. Mechanistic studies revealed an ERK_1/2_-dependent metabolic shift toward oxidative phosphorylation with concomitant suppression of glycolysis in FL-primed CAR-T cells. Importantly, this approach is readily translatable, GMP-compatible, and does not require genetic modification.

## Supplementary Material

Supplementary figures.

## Data Availability

The single-cell RNA-seq data from the previous study are publicly available at GEO database under accession GSE197268. The bulk RNA-seq raw data have been deposited in NCBI BioProject database under accession number PRJNA1422926. Analysis code is available upon reasonable request.

## Abbreviations

α-KG: α-ketoglutarate
BLI: bioluminescence
CAR: chimeric antigen receptor
ECAR: extracellular acidification rate
ELISA: enzyme-linked immunosorbent assay
FDR: false discovery rate
FL: fluvastatin
LC-MS: liquid chromatography-mass spectrometry
GFP: green fluorescent protein
HMGCR: 3-hydroxy-3-methylglutaryl-CoA reductase
IHC: immunohistochemistry
MSLN: mesothelin
NSCLC: non-small cell lung cancer
OCR: oxygen consumption rate
OXPHOS: oxidative phosphorylation
scRNA-seq: single-cell RNA sequencing
SEM: standard error of the mean
TCA: tricarboxylic acid
TEM: transmission electron microscopy
TME: tumor microenvironment
