# Supplementary material for: Redirecting Cholesterol Metabolism During Ex Vivo Biomanufacturing Enhances the Metabolic Fitness and Antitumor Efficacy of CAR-T Cells
Source: Int J Biol Sci. 2026 May 11;22(10):5228–45. doi: 10.7150/ijbs.132207 (PMC13215251; doi:10.7150/ijbs.132207)
Supplement: Supplementary file 1 — Supplementary figures. [file ijbsv22p5228s1.pdf]

Supplemental Material

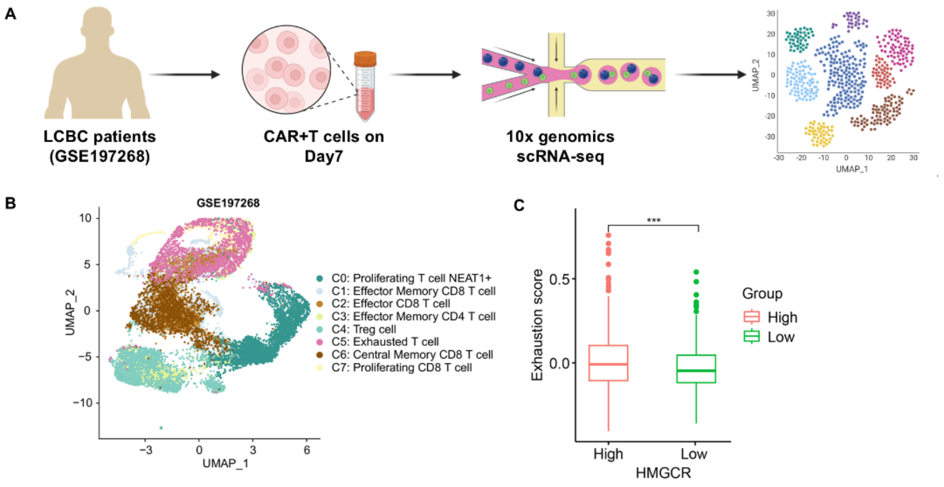

Figure S1. CAR-T cell exhaustion is closely associated with HMGCR expression.

(A) Schematic overview of the reanalysis pipeline for a publicly available scRNA-seq dataset of peripheral blood CD19 CAR-T cells from large B-cell lymphoma (LBCL) patients treated with CD19 CAR-T cell therapy. (B) UMAP plot of integrated scRNA-seq data showing distinct transcriptional clusters of CAR-T cells. (C) Boxplot comparing exhaustion signature scores between CAR-T cells with high versus low HMGCR expression levels. ****P* < 0.001.

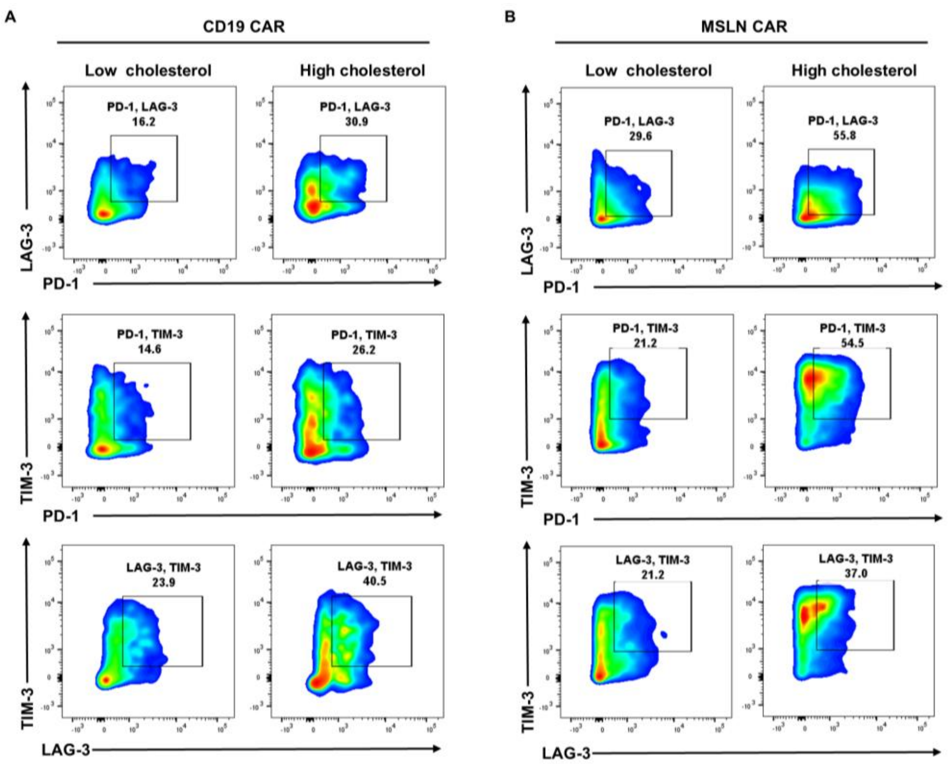

Figure S2. Representative flow cytometry plots of exhaustion marker co-expression in low- and high-cholesterol CAR-T-cell subsets.

Representative flow cytometry plots showing the co-expression of PD-1 and LAG-3, PD-1 and TIM-3, and LAG-3 and TIM-3 in low-cholesterol and high-cholesterol subsets of CD19 CAR-T cells (A) and MSLN CAR-T cells (B). CAR-T cells were divided into low- and high-cholesterol subsets based on Filipin III staining intensity.

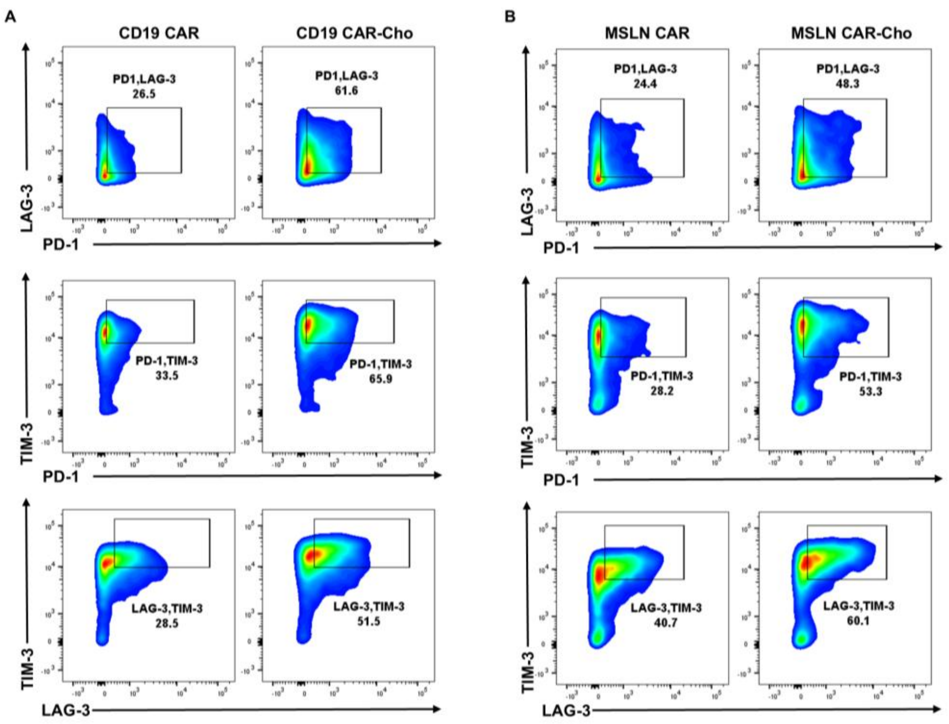

Figure S3. Exogenous cholesterol supplementation led to exacerbated exhaustion phenotypes.

Representative flow cytometry plots showing the co-expression of PD-1 and LAG-3, PD-1 and TIM-3, and LAG-3 and TIM-3 in control and cholesterol-treated CD19 CAR-T cells (A) and MSLN CAR-T cells (B).

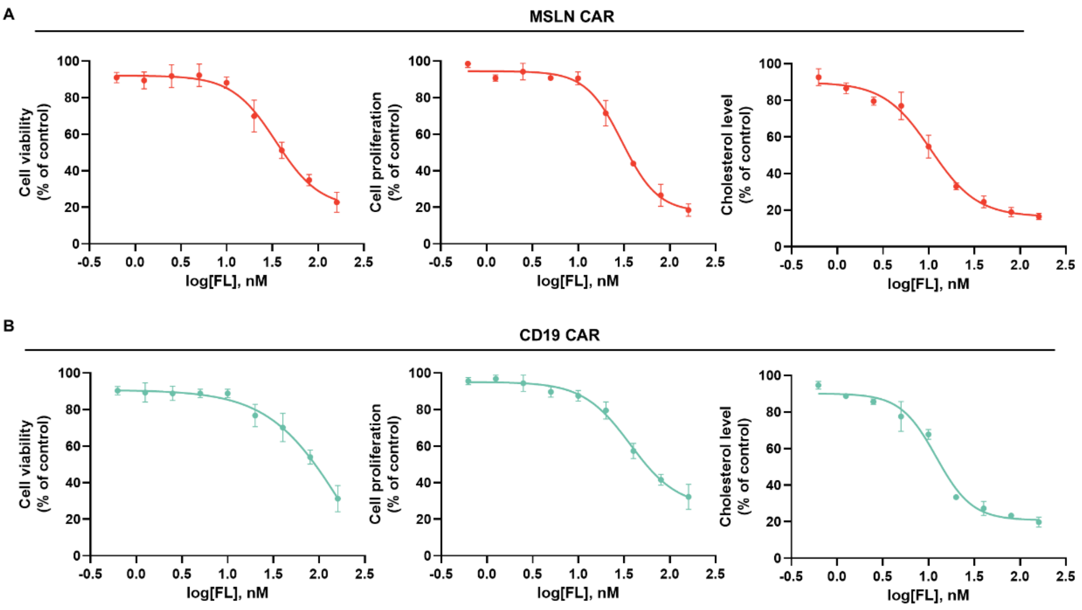

Figure S4. Effects of FL priming on CAR-T cell viability, proliferative capacity and cholesterol level.

During the *ex vivo* manufacturing of MSLN CAR-T cells (A) and CD19 CAR-T cells (B), the cells were treated with different concentrations of FL (0, 0.625, 1.25, 2.5, 5, 10, 20, 40, 80, and 160 nM) for 7 days (n=3). CAR-T cell viability and absolute number of viable CAR-T cells were measured, and cholesterol content was assessed by Filipin III staining. Dose–response curves were then generated after normalization to the control group, and were fitted by nonlinear regression using a four-parameter logistic model.

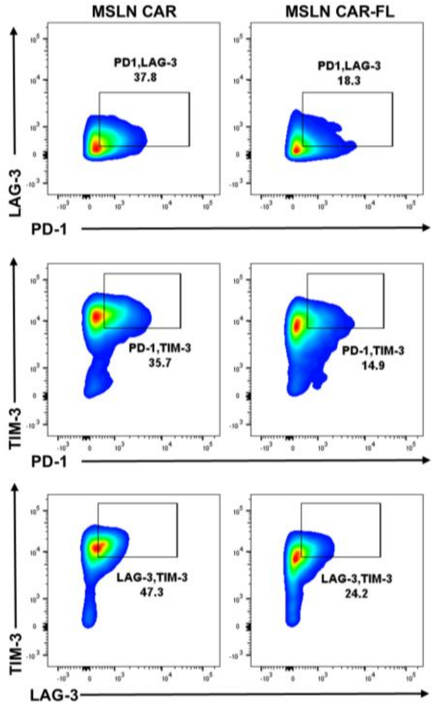

Figure S5. FL priming reduced the expression of exhaustion markers.

Representative flow cytometry plots showing the co-expression of PD-1 and LAG-3, PD-1 and TIM-3, and LAG-3 and TIM-3 in control and FL-primed MSLN CAR-T cells.

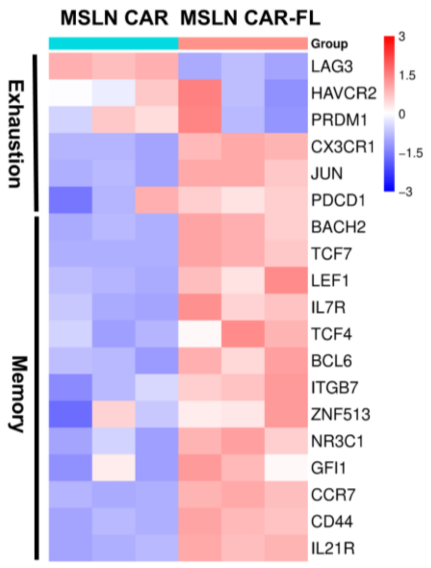

Figure S6. Heatmap of exhaustion- and memory-associated gene expression in MSLN CAR-T cells.

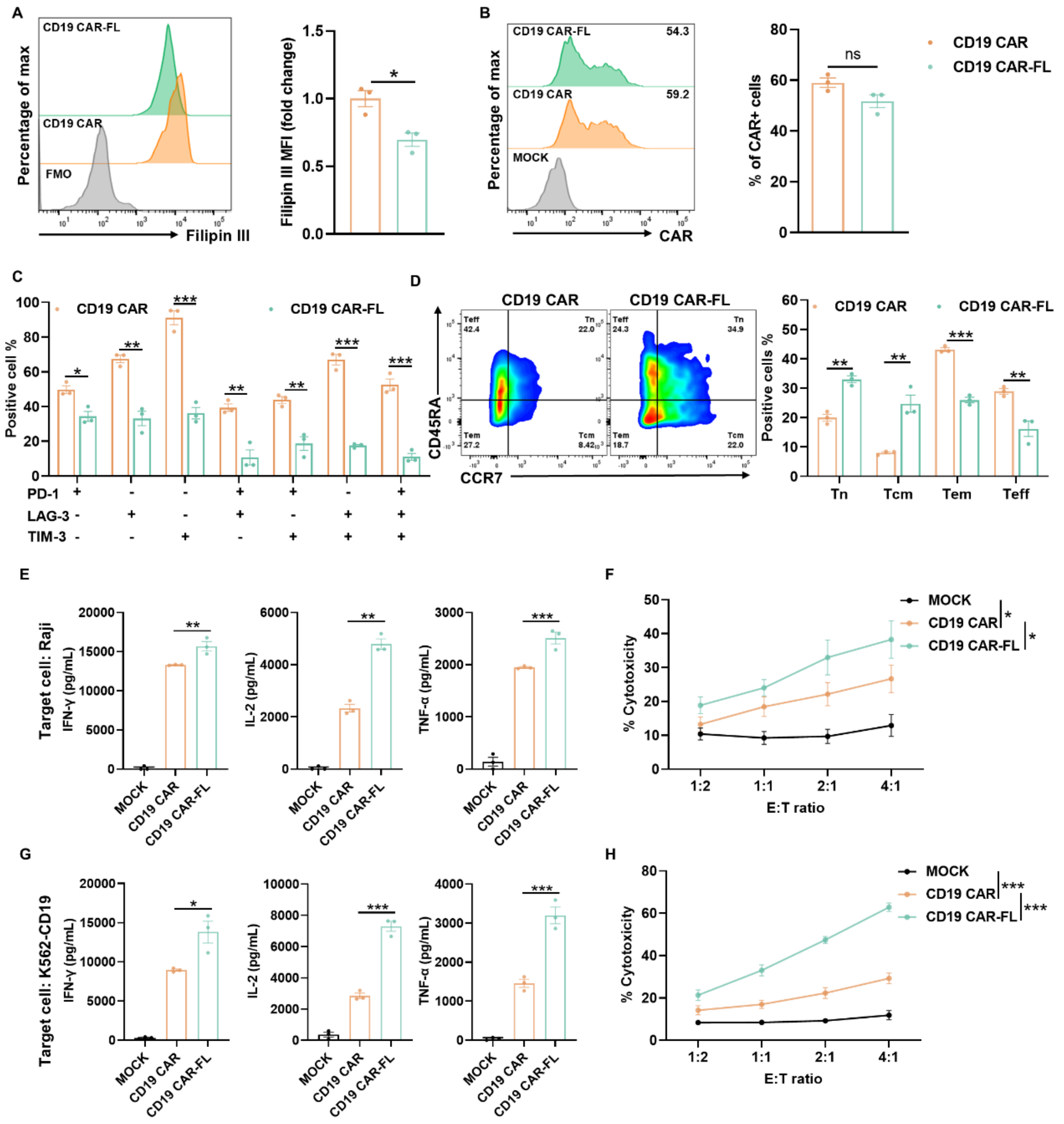

Figure S7. FL priming enhances phenotype and function of CD19 CAR-T cells.

(A) Representative histogram and quantification of Filipin III staining showing reduced cholesterol levels in CD19 CAR-T cells with FL treatment (n=3). (B) CD19 CAR surface expression in the presence or absence of FL, as measured by flow cytometry (n=3). (C-D) Phenotypic effects of FL priming on CD19 CAR-T cells. (C) Co-expression of PD-1, LAG-3, and TIM-3; (D) Memory phenotype based on CCR7 and CD45RA expression (n=3). (E, G) ELISA quantification of IFN-γ, IL-2, and TNF-α levels in supernatants of control or FL-primed CD19 CAR-T cells after 24-hour co-culture with Raji cells (E) or K562-CD19 cells (G) at an effector-to-target (E:T) ratio of 2:1 (n=3). (F, H) Cytotoxicity of control or FL-primed CD19 CAR-T cells against Raji (F) or K562-CD19 (H) cells at indicated E:T ratios, measured by CFSE/PI staining (n=3). Data are presented as mean ± SEM from three independent experiments. Statistical analysis was performed using unpaired two-tailed Student's t-test (A,

B), multiple Student's t-tests with FDR correction (Benjamini–Hochberg method) (C, D), one-way ANOVA (E, G) or two-way ANOVA (F, H), followed by Tukey's post hoc test. $*P < 0.05$, $**P < 0.01$, $***P < 0.001$.

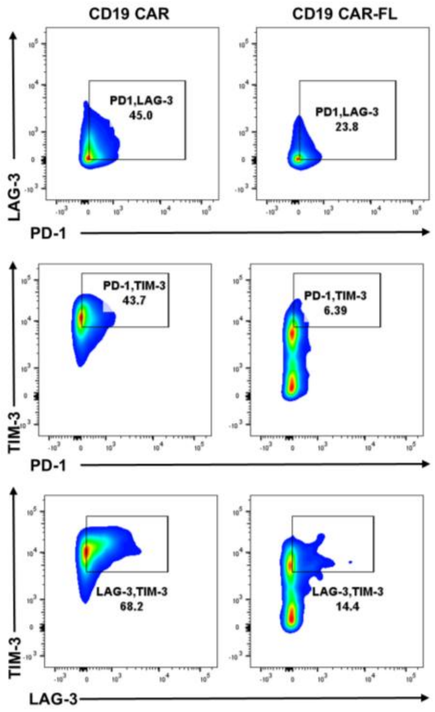

Figure S8. FL priming reduced the expression of exhaustion markers.

Representative flow cytometry plots showing the co-expression of PD-1 and LAG-3, PD-1 and TIM-3, and LAG-3 and TIM-3 in control and FL-primed CD19 CAR-T cells.

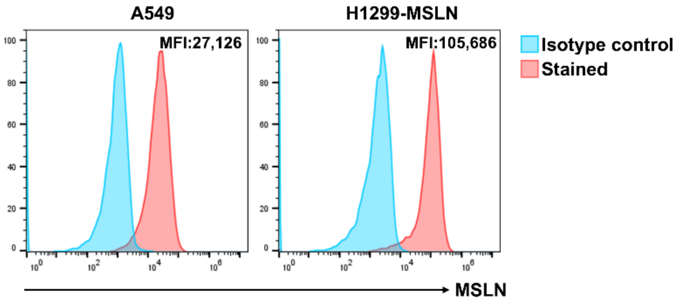

Figure S9. Flow cytometry analysis of MSLN expression on A549 and H1299-MSLN cells.

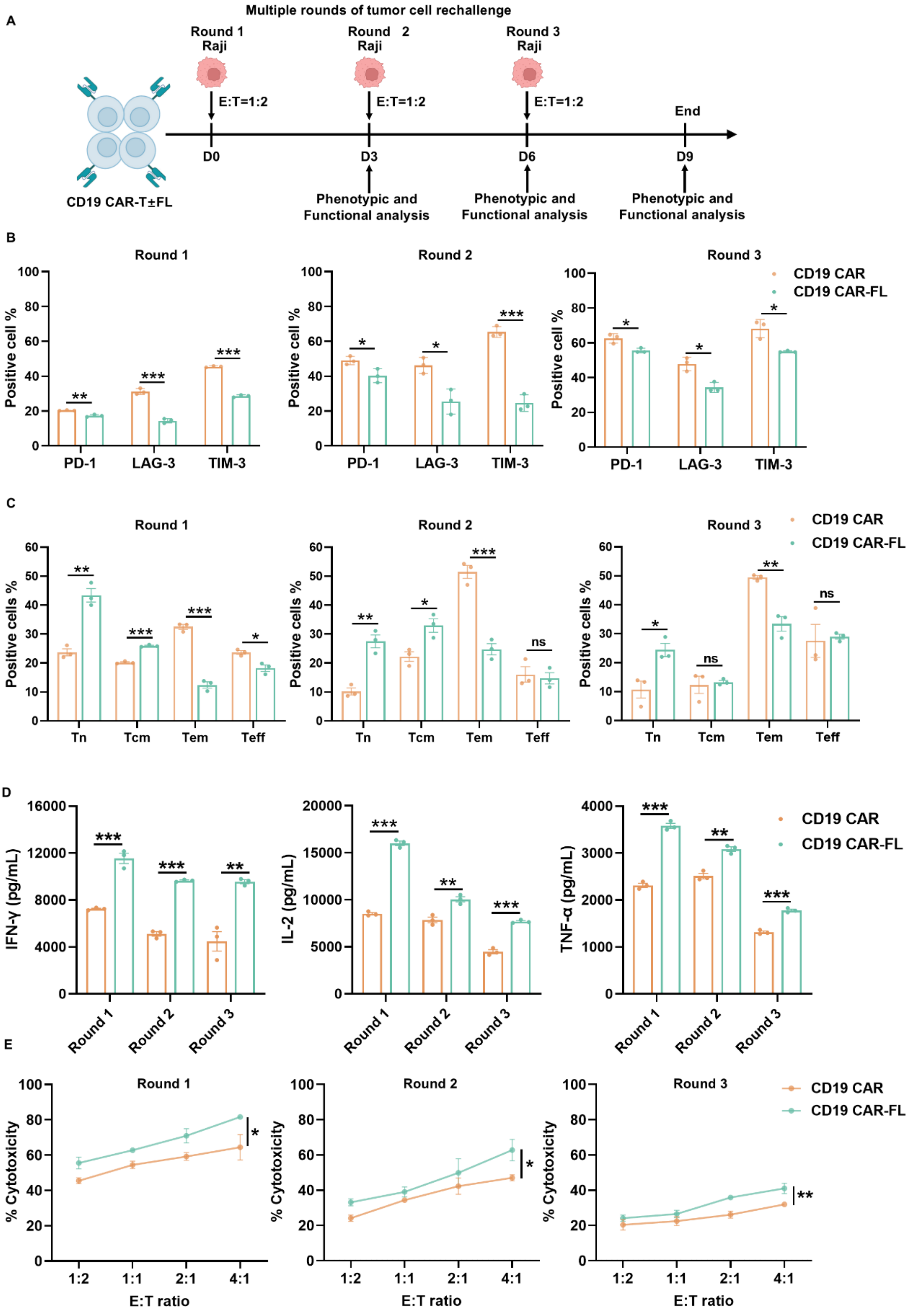

Figure S10. FL priming enhances the persistence of CD19 CAR-T cell anti-tumor activity upon serial tumor cell rechallenge.

(A) Schematic diagram of the serial tumor cell rechallenge experimental design. CD19 CAR-T cells with or without FL priming were subjected to multiple rounds of targeted tumor cell

rechallenge at an E:T ratio of 1:2 on days 0, 3, and 6, followed by phenotypic and functional analysis after each round of rechallenge on day 3, 6, and 9. (B-C) Phenotypic effects of FL priming on CD19 CAR-T cells upon serial tumor cell rechallenge. (B) The expression of PD-1, LAG-3, and TIM-3; (C) Memory phenotype based on CCR7 and CD45RA expression (n=3). (D) Cytokine secretion of CD19 CAR-T cells after each round of rechallenge. Concentrations of IFN-γ, IL-2, and TNF-α in culture supernatants were measured by ELISA (n=3). (E) Serial cytotoxicity assays of CD19 CAR-T cells against Raji. The cytotoxicity of CD19 CAR-T cells was assessed at indicated E:T ratios after each round of rechallenge, measured by CFSE/PI staining (n=3). Data are shown as mean ± SEM. Statistical significance was determined by multiple Student's t-tests with FDR correction (Benjamini–Hochberg method) (B, C, D), or two-way ANOVA with multiple comparisons (E). $*P < 0.05$, $**P < 0.01$, $***P < 0.001$; ns, not significant.

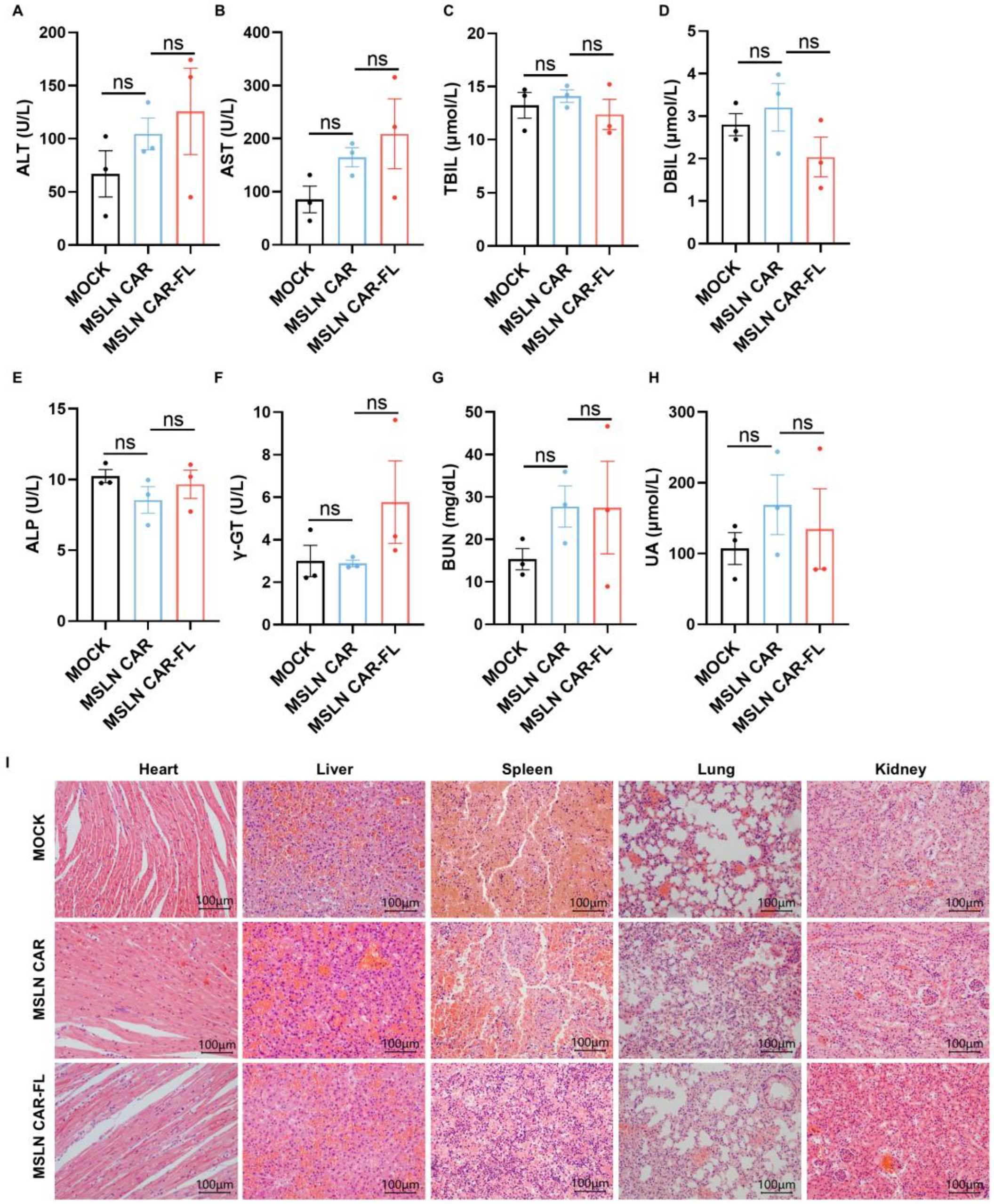

Figure S11. In vivo safety evaluation of FL-primed MSLN CAR-T cells.

Serum biochemical parameters were measured at the experimental endpoint to assess liver and kidney function in mice treated with MOCK, MSLN CAR, or MSLN CAR-FL cells. Levels of ALT (A), AST (B), TBIL (C), DBIL (D), ALP (E), γ-GT (F), BUN (G), and UA (H) are shown (n=3). (I) Representative HE staining images of the heart, liver, spleen, lung, and kidney from each group at the experimental endpoint (scale bars=100 μm). Statistical significance was determined using one-way ANOVA with Tukey's post hoc test (A-H). ns, not significant.

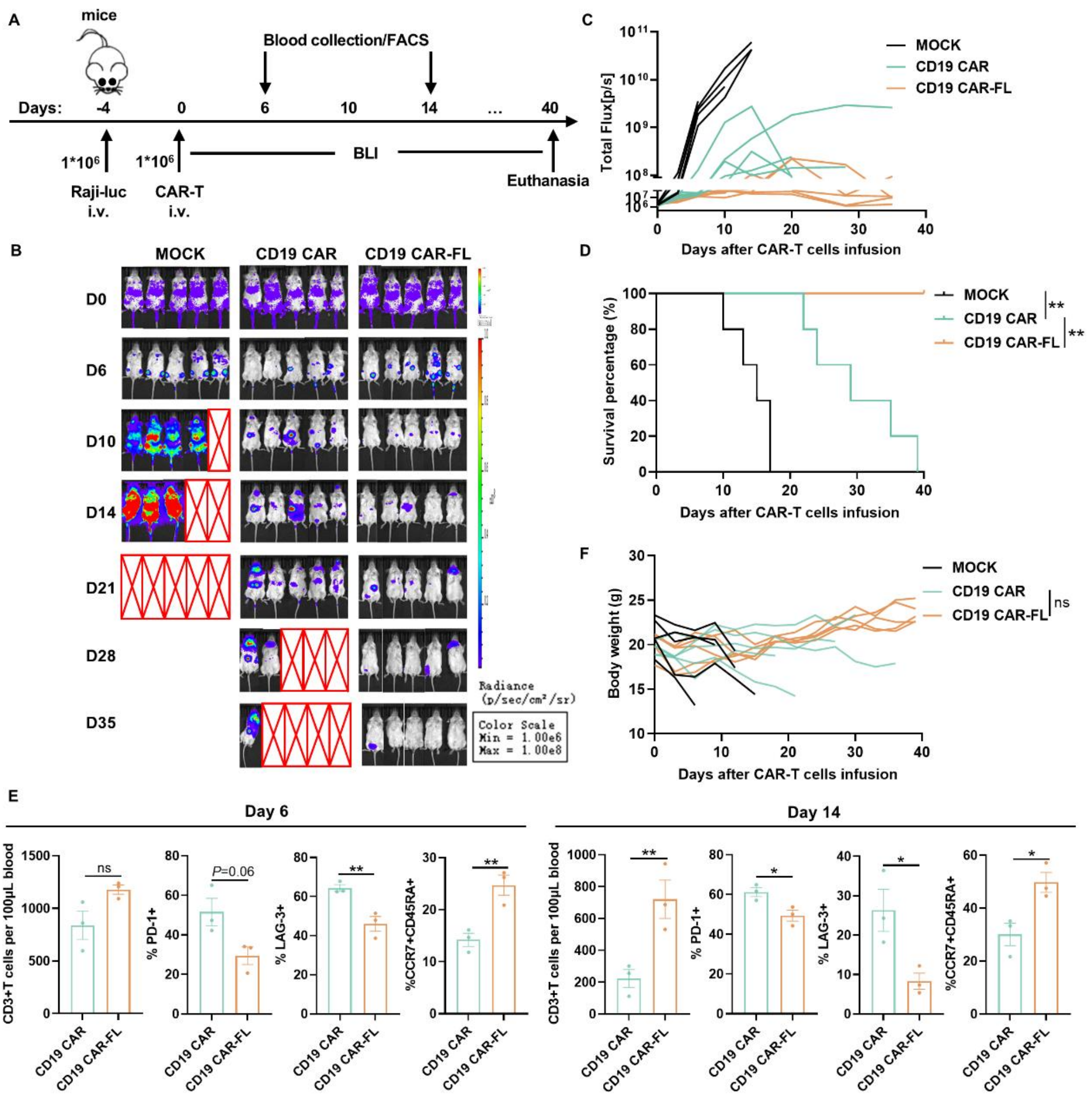

Figure S12. FL priming improves *in vivo* antitumor efficacy of CD19 CAR-T cells in a Burkitt lymphoma model.

(A) Experimental scheme: NCG mice were injected i.v. with Raji-luc cells (1×10⁶), followed by infusion of MOCK, control, or FL-primed CD19 CAR-T cells (1×10⁶) on day 0. Tumor burden was monitored via bioluminescent imaging (BLI); peripheral blood was analyzed on days 6 and 14. (B-C) Representative BLI images (B) and quantification of tumor burden over time (C) (n=5). (D) Kaplan–Meier survival curves of mice in each group (n=5). (E) Flow cytometry of peripheral blood T cells: CD3+ cell counts, exhaustion markers (PD-1, LAG-3) and naïve subset (CCR7+CD45RA+) on day 6 and 14 (n=3). (F) Longitudinal body weight measurements (n=5). Survival differences were analyzed using log-rank test (D), unpaired two-tailed Student's t-test (E), or two-way ANOVA with Tukey's post hoc test (F). *$P < 0.05$, **$P < 0.01$; ns, not significant.

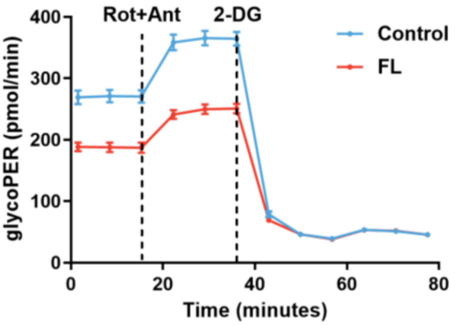

Figure S13. Glycolytic proton efflux rate (glycoPER) of the control and FL-primed MSLN CAR-T cells.

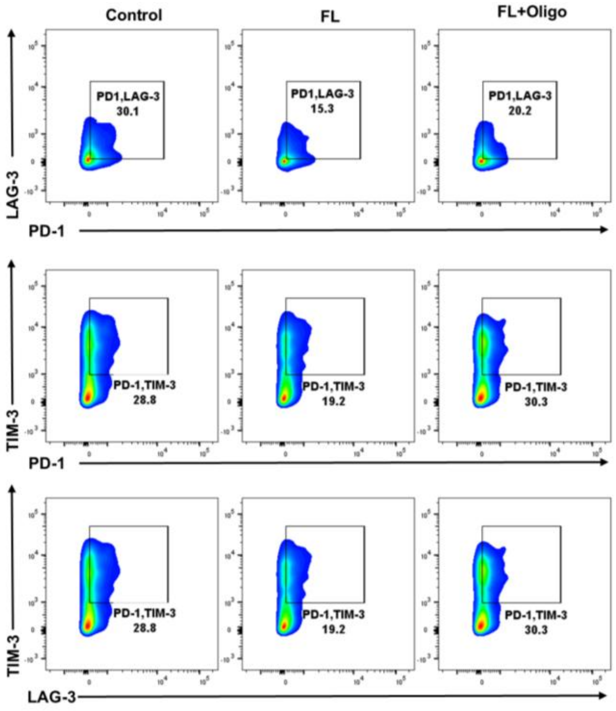

Figure S14. Oligomycin abrogated the effect of FL on reducing the exhausted phenotype.

Representative flow cytometry plots showing the co-expression of PD-1 and LAG-3, PD-1 and TIM-3, and LAG-3 and TIM-3 in control MSLN CAR-T cells, FL-primed MSLN CAR-T cells, and FL-primed MSLN CAR-T cells treated with 2.5 μM oligomycin for 3 days.
